# Effects of different exercise interventions on executive function in children with autism spectrum disorder: a network meta-analysis

**DOI:** 10.3389/fpsyt.2024.1440123

**Published:** 2024-09-13

**Authors:** Yaoqi Hou, Yan Wang, Jiaqi Deng, Xiangqin Song

**Affiliations:** College of Physical Education and Sports, Beijing Normal University, Beijing, China

**Keywords:** autism spectrum disorders, physical activity, network meta-analysis, executive functions, children

## Abstract

**Background:**

A large body of research has identified the positive effects of physical activity on children with autism spectrum disorders (ASD). However, the specific benefits of different types of sports on executive functioning in children with ASD remain unclear. The aim of this study was to further analyze the effects of different sports on executive functioning in children with ASD using reticulated meta-analysis and to establish their effectiveness ranking.

**Methods:**

This study conducted a comprehensive online search in Web of Science, PubMed, Cochrane, Embase, and CNKI databases. It included randomized controlled trials and quasi-experimental studies, and synthesized the data using a Bayesian framework.

**Results:**

Several relevant studies were included. The results showed that physical activity significantly improved all three dimensions of executive functioning (inhibitory control, cognitive flexibility, and working memory) in children with ASD. The improvement in cognitive flexibility and inhibitory control both reached a medium effect size. However, the improvement in inhibitory control was better than that in cognitive flexibility, while the improvement in working memory did not reach the level of a medium effect. Mini Basketball was effective in improving inhibitory control and cognitive flexibility, but not working memory. Ping Pong was more effective in cognitive flexibility and working memory, but weaker in inhibitory control. Fixed Bicycle was less effective in all three dimensions. Among other interventions, Learning Bicycles, Animal-assisted therapy, and Exergaming performed better in cognitive flexibility. SPARK, Neiyang Gong, and Martial Arts were also effective in improving inhibitory control. However, SPARK and Fixed Bicycle were not significant in improving working memory.

**Conclusion:**

Physical activity as an intervention can significantly improve the executive function of children with ASD. The intervention effects of different sports on different dimensions of executive function vary. Mini Basketball was outstanding in improving inhibitory control and cognitive flexibility. Ping Pong was effective in improving cognitive flexibility and working memory. Fixed Bicycle was not effective in any dimension.

## 1 Introduction

Autism Spectrum Disorder (ASD) is a neurodevelopmental disorder, with roughly 1 in 36 children identified as having ASD. The specific prevalence rates vary by region and age (1). Children with ASD often exhibit impaired social interactions (2), limited communication skills (3), and stereotyped behavioral patterns (4), These symptoms are usually seen before the age of three, which is also a critical period for children to develop cognition (5). Due to the complexity of interventions for autism spectrum disorders, many parents have to invest a great deal of time and energy, sometimes even choosing to quit their jobs to be fully responsible for their child’s intervention. This situation may lead them to focus all of their attention and resources on their children and neglect their own professional development, social life, and the needs of other family members, thus adversely affecting the overall family dynamics and the parents’ own physical and mental health. Behavioral problems and communication deficits in children with ASD can also lead to tensions and emotional distress among family members, increasing the risk of family splitting (6).

Executive functions are a collection of higher cognitive processes covering core functions such as planning, decision-making, error monitoring, emotion regulation, and behavioral inhibition (7). Children with autism spectrum disorders (ASD) typically have varying degrees of impairment in executive functioning, which in turn affects their ability to learn, self-care in daily life, and social behavior (8). Three main dimensions of executive functioning that are commonly discussed are inhibitory control, cognitive flexibility, and working memory (7). Inhibitory control refers to an individual’s ability to inhibit instinctive responses or behaviors; deficits in this area may lead to impulsive behaviors and social difficulties. Cognitive flexibility involves the ability to switch flexibly between different thoughts or behaviors; deficits in cognitive flexibility may manifest as discomfort and difficulty with change (9). Working memory is the ability to temporarily store and manipulate information; deficits in working memory may lead to difficulties in completing multi-step tasks and memory problems (10). These deficits affect many aspects of life and learning in children with ASD. Therefore, the adaptive capacity of children with ASD can be improved by enhancing executive functioning as a whole. In recent years, exercise has been recognized as an effective intervention to improve the development of psychological, physiological, and social functioning in children with autism spectrum disorders (ASD). A large number of empirical studies have shown that appropriate physical activity can significantly enhance executive functioning in various populations, including those with ASD or ADHD (11–13).

Limitations of the current study remain mainly in the following aspects: focusing on a single type of exercise intervention, such as swimming, running, or team sports, and the lack of studies that comprehensively compare the relative effects and adaptations of various types of exercise interventions. This limitation makes it difficult to differentiate between the effects of different types of exercise on executive function in children with ASD.

To further explore the effects of different sport interventions, meta-analyses can be used. Traditional meta-analyses provide a way of integrating the results of multiple studies and evaluating the effects of a single intervention. However, they usually do not allow for direct comparisons of the relative effects of multiple interventions. In contrast, network meta-analysis (NMA) is able to integrate comparisons of multiple interventions, even if these interventions were not directly compared in the original studies. NMA allows comparisons between different interventions to be made, enabling researchers to rank and recommend the effectiveness of multiple interventions (14, 15). Based on the above background, the present study used reticulated meta-analysis to systematically assess the effects of different exercise interventions on executive function in children with ASD.

## 2 Methods

### 2.1 Search strategy

The Cochrane Library, Embase, Web of Science, PubMed, CNKI, and other databases were searched using a combination of subject terms and free words based on “Patient Population, Interventions, Comparisons, Outcomes, and Study Designs” (PICOS). Experimental studies on the effects of exercise on executive function in children with ASD were searched from the time of library construction to May 20, 2024. Relevant reviews and meta-analyses were also referenced to manually supplement high-quality studies that were not initially found. The search strategy combined the term “Autism Spectrum Disorder” with variations of the terms “sports” (e.g., sport, athletics, athletic, exercise), and “child” (e.g., adolescent, minor, kid,boy,girl, young people). The detailed search strategy used for this network meta-analysis is available as **Supplementary Material**. Refer to the S1 file for the detailed search formula.

### 2.2 Inclusion and exclusion criteria

Inclusion Criteria: The PICOS framework (Participants, Interventions, Comparisons, Outcomes, and Study Design) was used to establish scientifically rigorous inclusion criteria.

Participants (P): The study was limited to children and adolescents between the ages of 3 and 18 years old, diagnosed with Autism Spectrum Disorder (ASD) according to the American Diagnostic and Statistical Manual of Mental Disorders (DSM-IV or DSM-5). Participants should not have a significant physical impairment.

Interventions (I): Interventions include, but are not limited to, physical activity, sports, organized sports games, team sports, and specially designed adaptive sports programs. These activities should aim to improve participants’ social interactions, communication skills, and physical fitness.

Comparison (C): The study should include both an experimental and a control group, ensuring no significant differences between the two groups in pre-experimental baseline data. The experimental design should allow for the assessment of changes before and after the intervention.

Outcome (O): The primary focus of the study is the improvement or change in executive functioning in children and adolescents with ASD. Assessment tools should include scales that specifically address executive function or other scales that address relevant subscales of executive function.

Study Design (S): The study should include types such as randomized controlled trials (RCTs), quasi-experimental studies, and others. It should clearly state the type of design, the experimental design, the calculation of the sample size, the randomization process, and the use of blinding to ensure the quality and reliability of the study.

Exclusion criteria: ① The outcome indicators were unclear, without extractable relevant data, or irrelevant to the selected outcome indicators. ② Conference papers, reviews, case studies, uncontrolled studies. ③ Subjects were suffering from multiple diseases at the same time.

### 2.3 Literature screening and data extraction

The initial screening of the literature was conducted by two researchers independently based on the inclusion and exclusion criteria. The researchers mainly reviewed the titles and abstracts of the literature, and identified and judged the content that was not relevant to the topic to be excluded. Then, the full text of the literature remaining after the initial screening was read in detail, and a second round of screening was conducted based on the inclusion and exclusion criteria to exclude literature that did not meet the inclusion criteria. During the screening process, if two researchers disagreed, a consensus was attempted through discussion. If there was still disagreement, a third researcher was assigned to make the final judgment.

After the screening was completed, relevant information was extracted and tallied from the selected literature, including (1) basic information about the literature (e.g., authors, country of authorship, and time of publication); (2) details of the experimental design (including diagnostic criteria for autism spectrum disorders (ASDs), age range of the participants, average age, sample sizes of the experimental and control groups, and gender ratios); (3) details of the physical activity program (type of activity, length and frequency of intervention, control condition); (4) assessment scales for executive and social functioning, as well as pre- and post-experimental means and standard deviations; and (5) Cochrane Quality Assessment of Literature indicators.

Considering that lower scores on some scales indicated better effects, while higher scores on other scales indicated better effects, in order to standardize the direction of the effect sizes, the results of those studies whose lower scores indicated better effects were adjusted by multiplying their means and standard deviations by -1 to ensure a consistent direction of effect with those studies whose higher scores indicated better effects.

### 2.4 Risk of bias evaluation of included studies

This study used the Cochrane Risk of Bias Assessment Tool to conduct a comprehensive quality review of selected randomized controlled trials (RCTs). The assessment tool covers several key aspects, including randomized sequence generation, allocation concealment, implementation of participant and implementer blinding, blinding manipulation of outcome assessment, completeness of outcome data, selectivity of reporting, and other forms of bias that may exist. Through the literature quality assessment, the aim is to ensure the accuracy and reliability of the research results, which in turn improves the overall quality of the research.

### 2.5 Statistical analysis

The reticulated meta-analysis in this study was performed using R software (version 4.3.2) and the gemtc package, paired with the JAGS program and the Bayesian Markov chain Monte Carlo (MCMC) algorithm, to analyze the data under the random effects model. Bayesian analysis was used for the network meta-analysis, with four Markov chains employed for each model. The number of iterations was set to 20,000, with the first 5,000 used for annealing to diagnose the degree of convergence of the model through Potential Scale Reduction Factors (PSRF). A PSRF value close to 1 suggests satisfactory model convergence.

Given the diversity of scale instruments in the selected literature, this study applied the standardized mean difference (SMD) and its 95% confidence interval (95% CI) to estimate the effect size. Based on Cohen’s classification of effect sizes, SMD values of ≥|0.8| indicate a large effect, ≥|0.5| to <|0.8| refer to a medium effect, ≥|0.2| to <|0.5| indicate a smaller effect, and <|0.2| represents a weak effect (16).

The analysis initially consisted of direct and indirect comparisons of the various exercise intervention methods by constructing network evidence plots to visualize the direct comparative relationships between the interventions. The thickness of the connecting lines in the graph is proportional to the number of studies directly compared, with intervention methods with large sample sizes indicated by larger dots. In forming the closed loop of the evidence network, the node-splitting technique was applied to test for consistency, and a p-value of more than 0.05 indicated good consistency between the exercise intervention methods. Interventions that were not directly compared were indirectly assessed by means of reticulated meta-analysis. By comparing different intervention methods two by two, the SUCRA value was calculated and accordingly the intervention effect was ranked, where a higher SUCRA value indicated a better intervention effect and a lower one a worse effect.

## 3 Results

### 3.1 Results of the literature search

**Figure 1:**
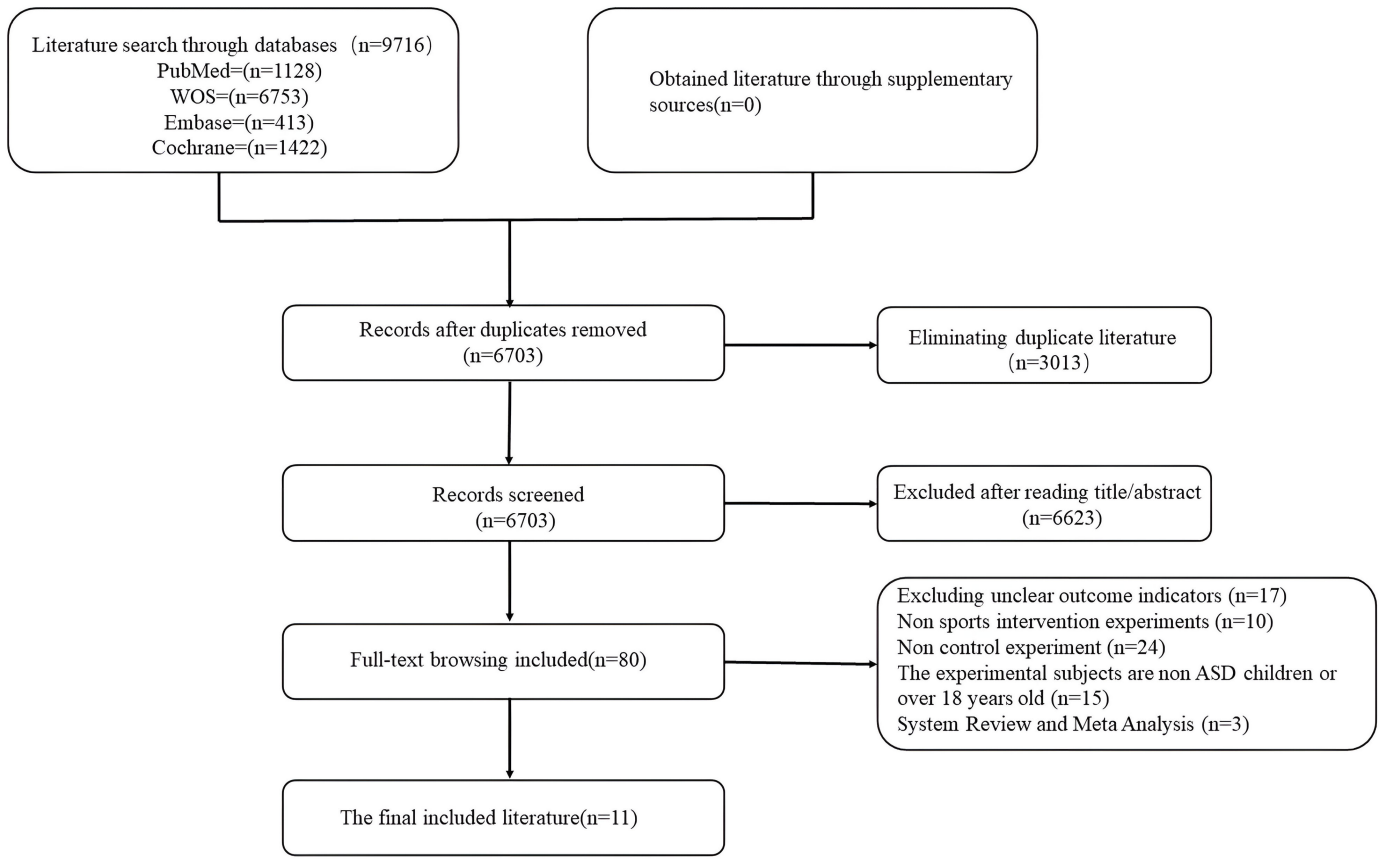
Flowchart of literature screening.

By searching multiple databases, a total of 9,716 documents were retrieved. These documents were included in Endnote for de-duplication, eliminating 3,013 duplicates and retaining 6,703 relevant documents. The titles and abstracts were then screened to exclude 6,623 irrelevant documents, resulting in 80 documents being initially included. The full texts of these 80 documents were retrieved and downloaded. Upon reading the full texts, 17 articles with unclear outcome indicators, 10 articles with non-sports intervention type experiments, 24 articles with non-controlled experiments, 15 articles with experimental subjects other than children with ASD or subjects older than 18 years old, and 3 articles with systematic reviews and meta-analyses were excluded. Finally, 11 relevant articles were included. The process of literature inclusion is shown in **Figure 1**.

The study included 11 papers involving 427 participants, with intervention cycles ranging from 2 to 13 weeks, intervention frequency ranging from 2 to 5 sessions per week, session durations ranging from 35 to 70 minutes, and total intervention durations ranging from 4 to 40 hours. Participants were aged between 3 and 18 years old, with a lower percentage of females. One study involved only male children and adolescents with ASD. The papers included in this study came from different countries: 7 from China, 1 from the United States, 1 from South Korea, 1 from Iran, and 1 from Italy.

The information table summarizes the first author’s name, year, country, age range and mean age of subjects, percentage of subjects who were female, experimental design, intervention period, and test scales of relevant indicators. Detailed information is shown in **Table 1**.

**Table 1:** Basic characteristics of the included studies.

Study	Country	Sample age (years)	Female percentage (%)	Design	Sample size (E/C)	Experimental group	Control group	Intervention dose	Outcome indicator	Outcome
Chan (2013) (17)	China	Mean:11.85 Range:6-17	10.00%	RCT	20/20	Neiyang Gong	Other sports	60min*2time*4week	ATEC CCTT-T2	CF
Wang (2020) (18)	China	Mean:4.91 Range:3-6	15.15%	NRCT	18/15	Mini Basketball	Conventional therapy	40min*5time*12week	SRS-2 CHEXI	IC/WM/CF
Borgi(2015) (19)	Italy	Mean:8.6 Range:6–12	0%	NRCT	15/13	Equestrianism	Conventional therapy	60min*1time*25weeks	TOL	CF
Liu(2023) (20)	China	Mean:4.79 Range:3-6	20.00%	RCT	15/15	Mini Basketball	Conventional therapy	40min*5time*12weeks	CHEXI	IC/WM
Nekar(2022) (21)	Korea	Mean:14.3 Range:6-18	9.09%	RCT	12/12	Exergaming	Conventional therapy	30min*2times*4weeks	Stroop	IC/WM/CF
Pan(2017) (22)	China	Mean:9.09 Range:6-12	0%	RCT	11/11	Ping Pong	Conventional therapy	70min*2times*12weeks	WCTS	IC/WM/CF
Phung(2019) (23)	United States	Mean:9.34 Range:8-11	21.00%	RCT	14/14	Martial Art	Conventional therapy	45min*2times*13weeks	Hearts Flowers BRIEF-2	IC/WM/CF
RafieiMilajerdi (2021) (24)	Iran	Mean:8.18 Range:6-10	5.17%	RCT	17/19/20	Exergaming/ SPARK	Conventional therapy	35min*3times*8weeks	WCST	IC/WM/CF
Tse(2023) (25)	China	Mean:9.94 Range:8-12	18.75%	RCT	23/19/22	Learning bicycles/ Fixed bicycle	Walk	60min*5times*2weeks	Stroop GNG	CF
Tse(2019) (26)	China	Mean:9.96 Range:8-12	6.45%	RCT	19/21	Mini Basketball	Conventional therapy	45min*2times*12weeks	GNG CBTT	IC/WM
Tse(2021) (27)	China	Mean:9.88 Range:8-12	24.00%	RCT	22/20/20	Learning bicycles/ Fixed bicycle	Conventional therapy	60min*5times*2weeks	TOL、GNG SCWT	IC/WM/CF

For convenience, only the first author is given. E/C means experimental group/control group; SRS-2, Social Responsiveness Scale; Second Edition; ATEC, Autism Treatment Evaluation Checklist; CCTT-T2, Children’s Color Trails Test-2; SRS-2, Social Responsiveness Scale; Second Edition; CHEXI, Childhood Executive Functioning Inventory; TOL, Tower of London; Stroop, Stroop Color and Word Test; WCTS, Wisconsin Card Sorting Test; Hearts Flowers, Hearts and Flowers Task; BRIEF-2, Behavior Rating Inventory of Executive Function; Second Edition; WCST, Wisconsin Card Sorting Test; GNG, Go/No-Go Task; CBTT, Corsi Block-Tapping Task; SCWT, Stroop Color and Word Tes.

### 3.2 Publication bias

There was some bias in the included literature, with most studies rated as “low” risk in terms of random sequence generation, indicating that these studies were of high quality in this area. However, in allocation concealment, blinding of participants and personnel, and blinding of outcome assessment, most of the studies were rated as “high” risk or no data (NA). This is because physical activity interventions are difficult to conduct with blinded subjects, so the main bias may come from the failure to double-blind. Detailed information is provided in **Table 2**.

**Table 2:** Quality evaluation of the included studies.

Trials	Random sequence generation	Allocation concealment	Blinding of participants and personnel	Blinding of outcome assessment	Incomplete outcome data	Selective reporting	Other bias
Borqi 2016 (19)	High	High	NA	NA	Low	Low	Low
Chan 2013 (17)	Low	Low	NA	NA	Low	Low	Low
Liu 2023 (20)	Low	Low	NA	NA	Low	Low	Low
Nekar 2022 (21)	Low	Low	Low	NA	Low	Low	Low
Pan 2017 (22)	Low	NA	High	NA	Low	Low	Low
Phung 2019 (23)	Low	NA	NA	NA	Low	Low	Low
Rafiei Milajerdi 2021 (24)	Low	NA	High	NA	Low	Low	Low
Tse 2019 (26)	Low	Low	NA	Low	Low	Low	Low
Tse 2021 (27)	Low	Low	NA	NA	Low	Low	Low
Tse 2023 (25)	Low	Low	NA	Low	Low	Low	Low
Wang 2020 (18)	High	High	NA	NA	Low	Low	Low

### 3.3 Results of data analysis

This reticulated meta-synthesis assessed the effects of different physical activities on executive functioning in children with autism spectrum disorders (ASD). The results showed that physical activity (PA) significantly improved all three dimensions of executive functioning in children with ASD. Improvements in cognitive flexibility and inhibitory control reached the medium effect level, with improvements in inhibitory control being better than those in cognitive flexibility. However, improvements in working memory from physical activity had not yet reached the medium effect level. The analysis suggests that physical activity can significantly improve executive functioning in children with ASD.

Inconsistency network modeling was used to simultaneously test the overall consistency of the direct and indirect effects for both pairwise and multi-arm comparisons, except for the working memory dimension which could not be tested for loop inconsistency due to insufficient closed loops. The results showed no significant overall inconsistency for inhibitory control, cognitive flexibility, or both dimensions. Sensitivity analysis scores revealed that the significance of the results was not significantly affected by variation in study quality. In addition, there was no significant publication bias when comparing the adjusted funnel plots (**Appendix 2**).

In this study, the variables of year of inclusion, age of subjects, male/female ratio of subjects, weekly intervention frequency of physical activity, total intervention duration period, single intervention duration, and total dry hours were included in the meta-regression to analyze the results. It was found that none of the above-mentioned factors showed significant moderating effects, which indicated that the regression hypothesis was valid (**Appendix 2**).

#### 3.3.1 Effectiveness of physical activity on cognitive flexibility interventions for children with autism spectrum disorders

**Figure 2:**
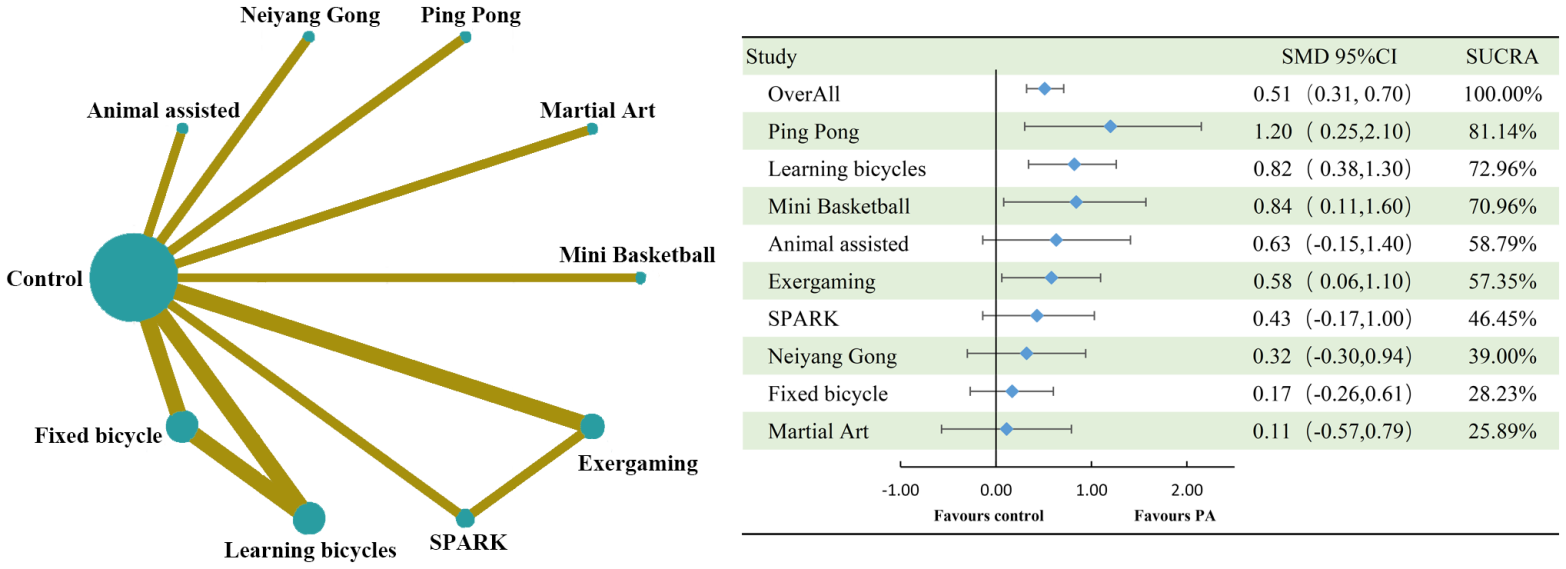
Comparison of cognitive flexibility dimensions of executive function and network diagram of therapeutic effects between different treatments compared to the control group. PA, physical activity; SMD, standardized mean deviation; SUCRA, the cumulative ranking curve below the surface.

**Figure 3:**
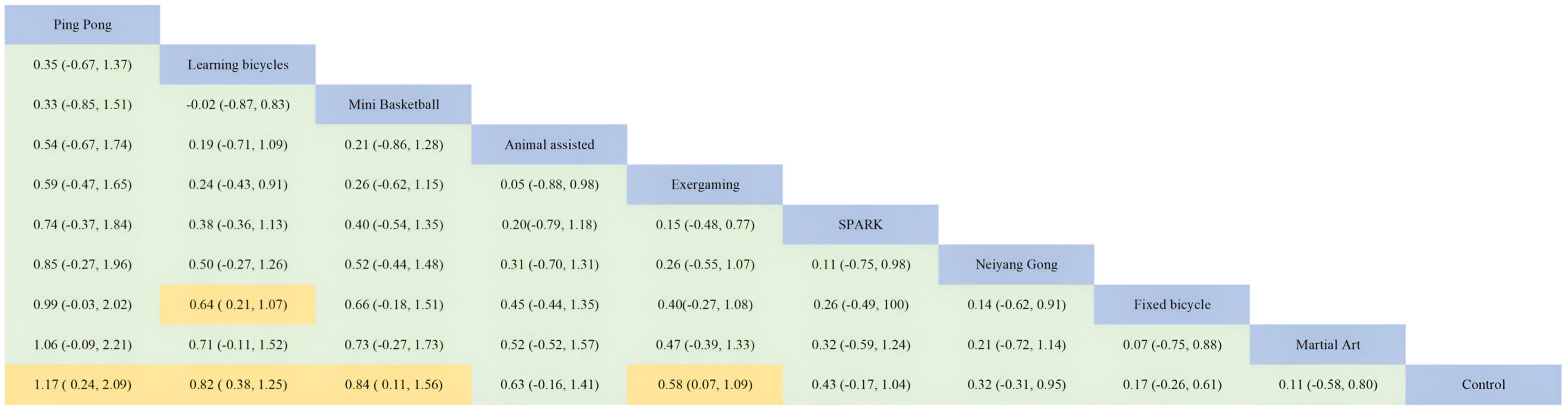
Ranking of results analysis for functional cognitive flexibility dimension.

The cognitive flexibility dimension involved nine interventions, three of which reached large effect levels two of which reached medium effect levels, two of which were in the small effect level range, and two of which were at the slight effect level. The overall effect size was at a medium level (SMD=0.60, 95%CI: 0.41 to 0.78). Ping Pong (SMD=1.20, 95%CI: 0.25 to 2.10, SUCRA: 81.14%) did not have a very prominent intervention effect in the inhibitory control dimension but had the highest effect in the cognitive flexibility dimension, followed by Learning bicycles (SMD=0.82, 95%CI: 0.38 to 1.30, SUCRA: 72.96%, SUCRA: 72.96%). SUCRA: 72.96%), Mini Basketball (SMD=0.84, 95%CI: 0.11 to 1.60, SUCRA: 70.96%) and Animal assisted (SMD=0.63, 95%CI: -0.15 to 1.40, SUCRA: 58.79%), Exergaming (SMD=0.58, 95%CI: 0.06 to 1.10, SUCRA: 57.35%), and the intervention effects of the remaining interventions SPARK (SMD=0.43, 95%CI: -0.17 to 1.00, SUCRA: 46.45%), Neiyang Gong (SMD= 0.32, 95%CI: -0.30 to 0.94, SUCRA: 39.00%), Fixed bicycle (SMD=0.17, 95%CI: -0.26 to 0.61, SUCRA: 28.23%), Martial Art (SMD=0.11, 95%CI: -0.57 to 0.79. SUCRA: 46.45%) None of the intervention effects were ten outstanding, where Martial Art ranked 3rd in all-time control in cognitive flexibility but ranked last, which may be due to. Detailed information is shown in **Figures 2** and **3**.

#### 3.3.2 Effectiveness of physical activity on inhibitory control interventions for children with autism spectrum disorders

**Figure 4:**
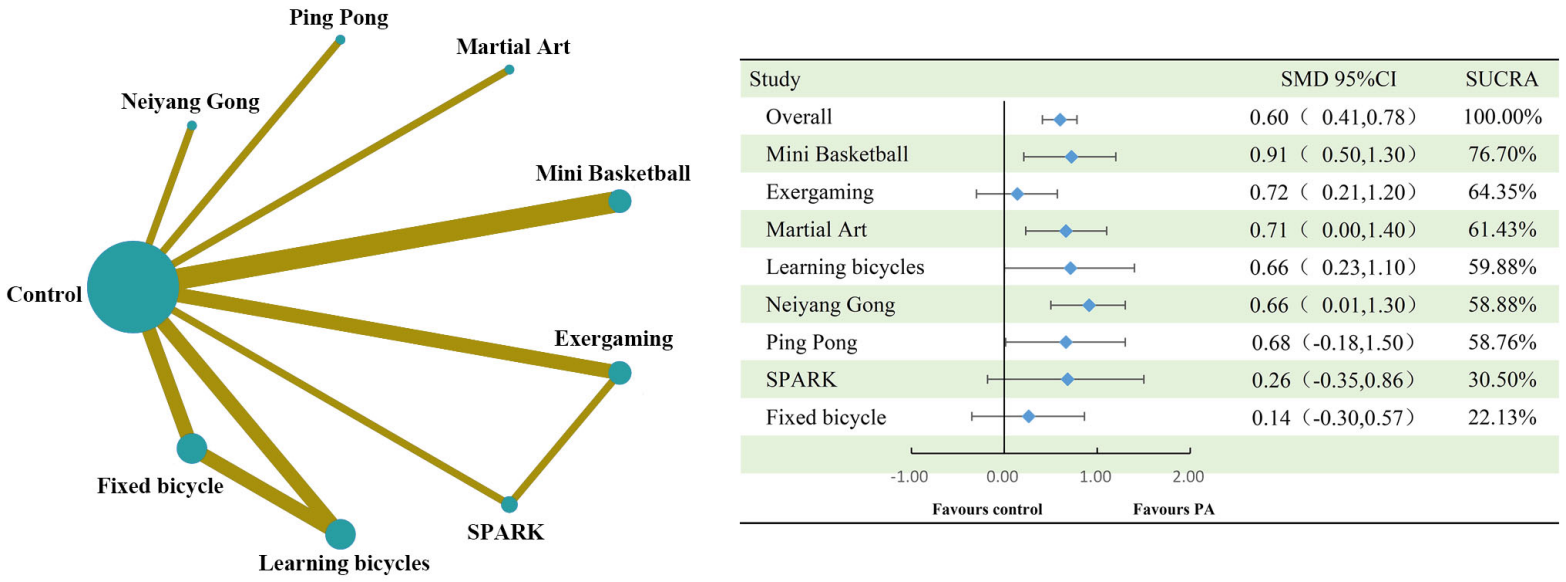
Compares the dimensions of executive function inhibition control and the network diagram of efficacy between different treatments compared to the control group. PA, physical activity; SMD, standardized mean deviation; SUCRA, the cumulative ranking curve below the surface.

**Figure 5:**
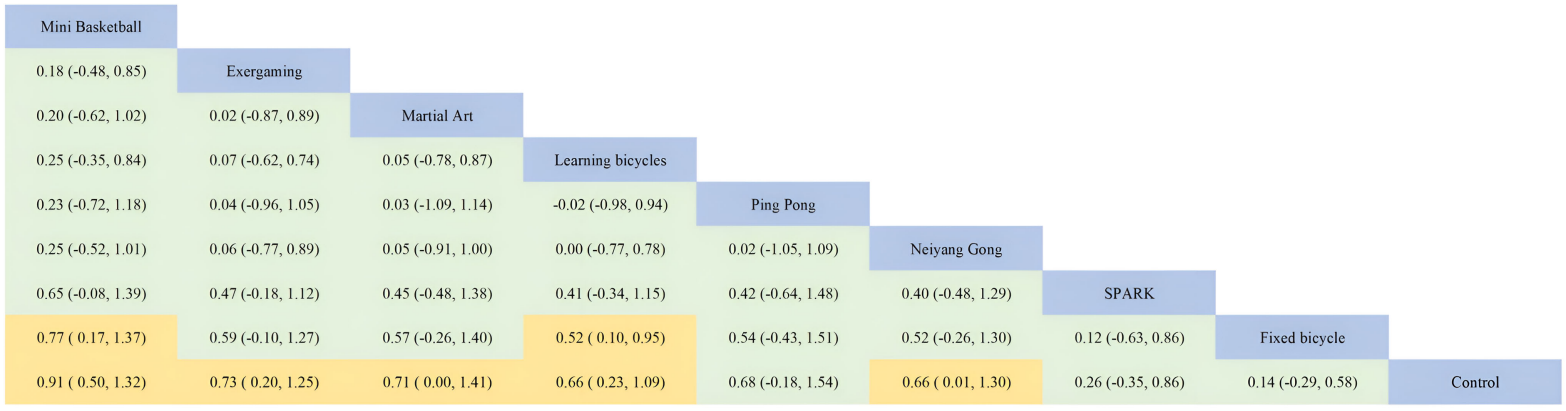
Ranking of results analysis for functional inhibition control dimension.

The results of the analysis showed that physical activity significantly improved inhibitory control in children with ASD and was slightly above the medium effect level (SMD=0.60, 95%CI: 0.41 to 0.78). Of the eight physical activities involved in the study, the Mini Basketball intervention had the largest effect size, reaching the large benefit level (SMD=0.91, 95%CI: 0.50 to 1.30, SUCRA: 76.70%). This was followed by Exergaming (SMD=0.72, 95%CI: 0.21 to 1.20, SUCRA: 64.35%), Martial Art (SMD=0.71, 95%CI: 0.00 to 1.40, SUCRA: 61.43%), Learning Bicycles (SMD=0.66, 95%CI: 0.23 to 1.10, SUCRA: 59.88%), Neiyang Gong (SMD=0.66, 95%CI: 0.01 to 1.30, SUCRA: 59.88%), and Ping Pong (SMD=0.68, 95% CI: -0.18 to 1.50, SUCRA: 58.76%), all of which were also above the medium effect level. However, SPARK (SMD=0.26, 95%CI: -0.35 to 0.86, SUCRA: 30.50%) only reached the small effect level, and Fixed Bicycle (SMD=0.14, 95%CI: -0.30 to 0.57, SUCRA: 22.13%) was even lower at the small effect level. See **Figures 4** and **5** for detailed information.

#### 3.3.3 Effectiveness of physical activity on working memory interventions for children with autism spectrum disorders

Compared to the other two dimensions, there were fewer types of interventions involving working memory, with only seven physical activities. Among these, two reached large effect levels, one was at a medium effect level, three were at a small effect level, and one showed no significant improvement. The overall effect level was close to medium.

**Figure 6:**
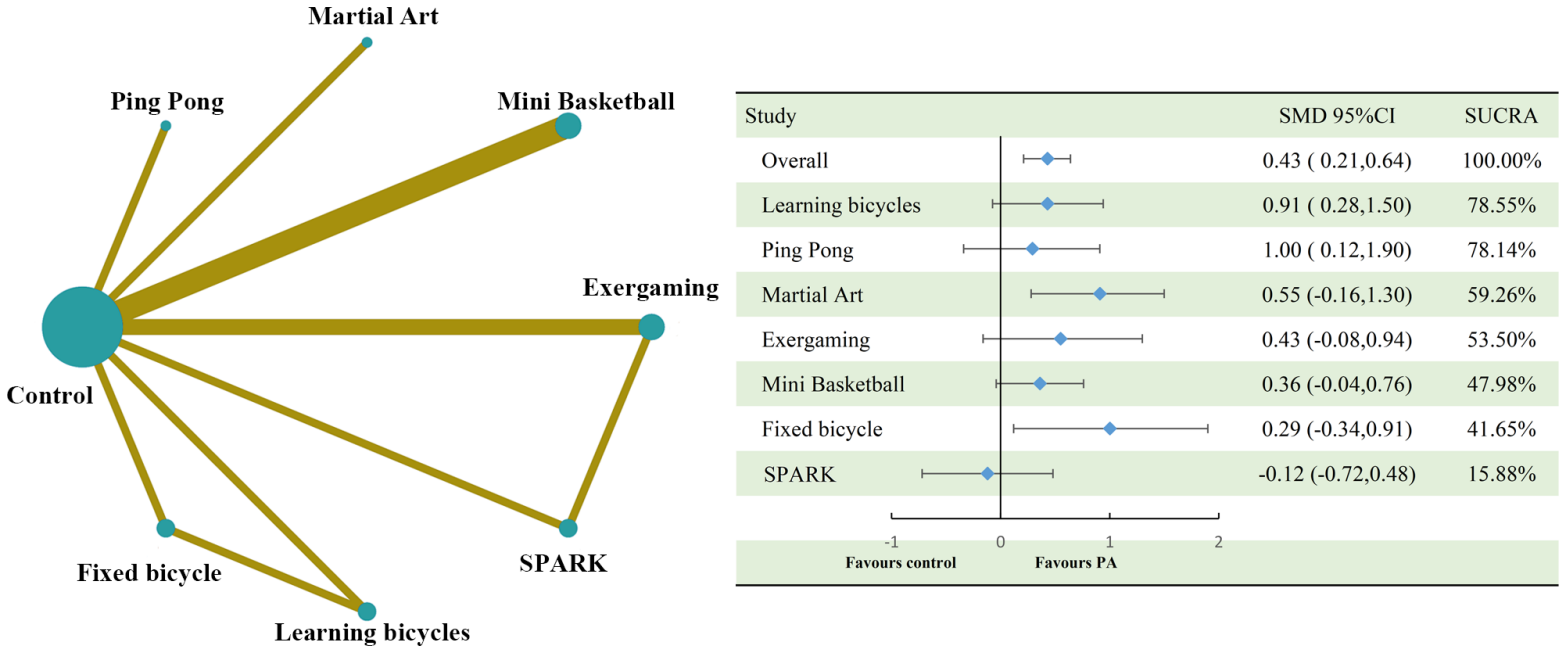
Comparison and efficacy network diagram of different treatments on the dimensions of executive functional working memory compared to the control group. PA, physical activity; SMD, standardized mean deviation; SUCRA, the cumulative ranking curve below the surface.

**Figure 7:**
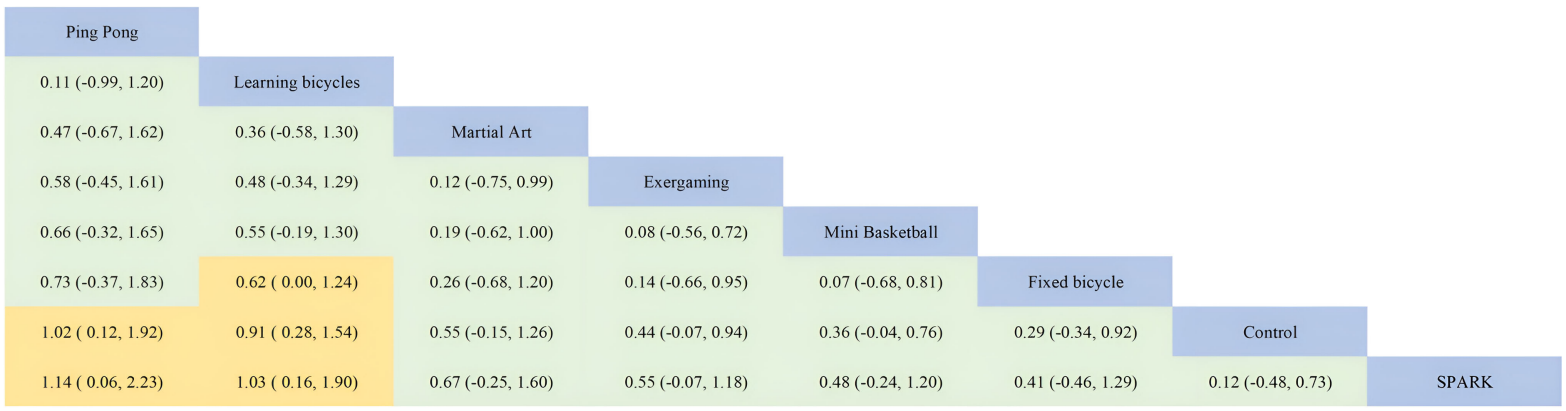
Ranking of results analysis for functional working memory dimension.

Ping Pong (SMD=1.00, 95%CI: 0.12 to 1.90, SUCRA: 78.14%) had the highest effect size, but its SUCRA value ranked second. Learning Bicycles (SMD=0.91, 95%CI: 0.28 to 1.50, SUCRA: 78.55%) ranked the highest in terms of SUCRA. Fixed Bicycle (SMD=0.29, 95%CI: -0.34 to 0.91, SUCRA: 41.65%) continued to perform poorly in working memory, and it is worth noting that this intervention performed relatively poorly on all three dimensions of executive functioning. Additionally, the SPARK exercise program showed a lack of significant improvement effect on working memory. Detailed information is shown in **Figures 6** and **7**.

## 4 Discussion

In this study, a loop inconsistency test was used to assess the consistency of results across different studies. The loop inconsistency test evaluates consistency by detecting circular comparisons between studies. The results showed no significant inconsistency in the dimensions of inhibitory control and cognitive flexibility, indicating good consistency of these results across different studies. However, due to the insufficient number of included studies, it was not possible to perform the loop inconsistency test for the working memory dimension. These findings suggest that the effects of different types of exercise interventions on improving executive function in children with ASD are consistent in the dimensions of inhibitory control and cognitive flexibility, further validating the reliability of the study results.

From the perspective of cognitive load theory, the high dynamism of Mini Basketball may increase cognitive load, thereby limiting the improvement of working memory capacity, as excessive cognitive load can inhibit effective information encoding and storage processes (28). Additionally, while Ping Pong has shown positive intervention effects on cognitive flexibility and working memory, its effect on inhibitory control is relatively poor. This could be because Ping Pong emphasizes rapid adaptability and memory strategies, aiding ASD children in these cognitive skills. However, the sport also requires quick reactions, and the fast-paced game rhythm and continuous stimulus-response do not provide enough time for participants to practice and develop the ability to resist impulsive responses (29). Mini Basketball shows the best intervention effect on inhibitory control and performs well in cognitive flexibility (ranking third), but its effect on working memory is not particularly outstanding (ranking second to last). The emphasis on quick decision-making and immediate response in Mini Basketball aligns with the cognitive needs of children with ASD (30). However, Mini Basketball lacks targeted memory exercises and sustained attention training components. Interestingly, not all sports can lead to improvements. The repetitive exercise of Fixed Bicycle scores low in all three dimensions. As a primarily aerobic activity, Fixed Bicycle lacks the dynamic interaction and social engagement present in other activities like Martial Arts or Mini Basketball. Compared to more diverse or integrated physical interventions, Fixed Bicycle is relatively monotonous, which may not sufficiently stimulate the development of multiple executive function domains such as inhibitory control, cognitive flexibility, and working memory in children with ASD. Consequently, it is less effective in improving executive function and fails to capture the interest of ASD children (31).

The results of the present study are consistent with previous literature in the core finding that physical activity significantly improves executive functioning in children with autism spectrum disorders (ASD) (32–34). Many studies have extensively confirmed the positive effects of exercise interventions on children with ASD, but the physiological mechanisms remain less explored. Studies have shown that the main cause of ASD symptoms is abnormal changes in synaptic structure and function (35). Williams found that the spine morphology on the dendrites of pyramidal neurons in adolescents and adults with ASD was significantly reduced in density, although it remained normal (36). However, another study found that cortical pyramidal neuron spine density was higher in patients with ASD (37), while Tang et al. noted that dendritic spine density of temporal lobe layer V pyramidal neurons in patients with ASD exceeded that of healthy controls (38). The reduced rate of spine reduction from childhood to adolescence suggests that there may be a defect in synaptic pruning in ASD, and that high-density spines are more prevalent in individuals with ASD who have more severe cognitive impairment (39). Abnormal changes in spine may lead to disruption of specific neural circuits (35). Exercise interventions can alter synaptic function and promote behavioral improvements by modulating structural plasticity of synapses and spines (40).

A 2022 study found higher levels of presynaptic proteins in individuals who regularly participated in physical activity in later life, suggesting a facilitative effect of exercise interventions on synaptic function (41). A mouse model study by Chen et al. showed that chronic plank exercise enhanced synaptogenesis, synaptic transmission, neuronal activity, and axonal myelin formation, thereby improving motor learning (42). Andoh et al. found that exercise intervention improved synaptic pruning deficits and abnormal behavior in ASD mice (43). Microglial cells are able to recognize the intensity of synaptic activity, and exercise intervention promotes healthy synapses by selectively removing weaker synapses. The complement molecule C1q and its downstream component C3 label inactive synapses, and the C3 receptor CR3 recognizes and prompts microglial cells to phagocytose these synapses (44). Synaptic responses to motor interventions may be related to the neurotrophic factor BDNF, which is enriched in the hippocampal mossy fiber region and is closely associated with learning and memory processes (45). Studies have shown that exercise intervention improves structural plasticity of synapses and spines by modulating the BDNF/TrkB signaling pathway (46–48).

Exercise influences synaptic plasticity and the BDNF/TrkB signaling pathway through various molecular mechanisms. During physical activity, neuronal excitability in the brain increases, leading to greater release of BDNF. This enhances certain synapses and promotes synaptic competition, thereby improving cognitive and executive functions and alleviating symptoms in children with ASD (49). BDNF activates TrkB receptors, triggering multiple signaling pathways such as Ras/ERK, PI3K, and PLC-γ. This regulation leads to local protein synthesis at synapses, thereby promoting synaptic plasticity (50). BDNF rapidly translocates TrkB receptors from non-raft regions to cholesterol-rich lipid rafts, enhancing synaptic transmission efficiency (51). In the hippocampus, BDNF enhances the mRNA levels of CREB and synapsin I, regulating gene transcription and synaptic transmission. This mediates the effects of exercise on synaptic plasticity (52). BDNF achieves local and synapse-specific regulation by promoting the selective expression of TrkB receptors at active synapses (53). Exercise positively impacts synaptic structure and function through the BDNF/TrkB signaling pathway. Under TrkB receptor activation, BDNF promotes neuronal growth and the formation and pruning of synapses, enhancing brain synaptic plasticity. These neurobiological mechanisms play a crucial role in improving executive function in children with ASD. By increasing BDNF levels, exercise activates TrkB receptors and their downstream signaling pathways, enhancing synaptic transmission and neural plasticity, thereby improving the cognitive function and behavioral performance of children with ASD.

Different types of exercise significantly impact neuroplasticity and function through the BDNF/TrkB signaling pathway. Studies have found that under conditions of complex motor learning and moderate exercise, the expression of BDNF and TrkB in the cerebellum and motor cortex of rats significantly increases. This effect is particularly lasting under conditions of complex motor learning (54). Aerobic training can increase BDNF levels and peripheral blood flow response (55). Additionally, moderate-intensity running combined with an enriched environment significantly enhances learning and memory abilities in rats and upregulates the expression of BDNF and TrkB. This further demonstrates that the BDNF/TrkB signaling pathway plays a regulatory role in the improvement of cognitive function through exercise and enriched environments (56). Mini Basketball, as an aerobic exercise involving frequent running and shooting, significantly increases BDNF levels in the brain. By enhancing the BDNF/TrkB signaling pathway, Mini Basketball can promote synapse formation and improve synaptic transmission efficiency, thereby improving cognitive flexibility and inhibitory control in children with ASD. In contrast, Ping Pong involves cognitive engagement, which continuously stimulates cognitive regions of the brain, promoting synaptic plasticity, enhancing synaptic transmission, and neuronal activity. This helps improve cognitive flexibility and working memory in children with ASD.

This study refines the comparison of intervention methods, systematically analyzing the effects of different physical activities on various dimensions of executive function in children with ASD. Using a network meta-analysis, the study confirms the positive impact of physical activities and identifies which specific types of activities excel in improving particular executive functions. Previous studies often focused on single intervention types, such as aerobic exercises or team sports, assessing their overall cognitive or behavioral benefits. These studies rarely directly compared multiple exercise forms or analyzed different executive function dimensions in detail.

Despite providing a systematic analysis of the impact of various physical interventions on the executive functions of children with ASD, this study has limitations. First, the limited number of included studies may affect the comprehensiveness and representativeness of the results. Although network meta-analysis allows for the comparison of multiple interventions, its outcomes are still constrained by the quality and detail of the original studies. Some studies may have selective reporting bias or incomplete data, potentially impacting the accuracy and reliability of the findings. For instance, some studies might not report all negative results, leading to an overestimation of the intervention effects.

Additionally, this study mainly relies on quantitative measures to assess intervention effects, lacking detailed observations of individual differences and intervention processes. This approach may overlook the personalized responses and needs of children with ASD during different physical interventions, limiting a comprehensive evaluation of the interventions’ actual effectiveness. While the study examines the effects of various interventions on inhibition control, cognitive flexibility, and working memory, the interplay between these dimensions and their overall impact on executive function requires further research. The current study design may not fully capture these complex relationships.

To overcome these limitations, future research should consider the following points: First, the number and quality of included studies should be increased to enhance the comprehensiveness and representativeness of the results. Future studies should include more high-quality randomized controlled trials to reduce issues of selective reporting bias and incomplete data.Second, detailed observations of individual differences and intervention processes should be increased to better understand the specific effects of different interventions on various children with ASD. This can be achieved through mixed-method research designs that combine quantitative and qualitative data collection and analysis, providing a more comprehensive evaluation of intervention effects.

Future research should explore more personalized and diverse physical interventions to meet the varying needs of children with ASD. Considering their sensory and cognitive needs, multisensory physical interventions combining visual, auditory, and tactile stimuli, such as sensory integration training, could help them better adapt to their environment. To enhance social skills and teamwork awareness, social interaction physical activities like group sports and cooperative games can be designed, which not only improve executive function but also promote social adaptation. Strengthening parent-child relationships through family-involved physical interventions, such as parent-child yoga and family gymnastics, can enhance family support systems. Increasing engagement and motivation with virtual reality and interactive games, such as VR games and interactive videos, can provide enjoyable and challenging physical experiences, thereby enhancing intervention effects. Implementing these personalized and diverse physical interventions can create a more comprehensive support system for the growth and development of children with ASD. This study employed a network meta-analysis to compare and quantify the effects of various physical interventions, providing a comprehensive perspective on their specific impact on the executive function of children with ASD. By comparing the effects of different interventions on cognitive flexibility, inhibition control, and working memory, this study offers specific clinical application recommendations and scientific evidence for future treatment strategies.

## 5 Conclusion

In summary, the present study assessed the effects of different physical activities on executive functioning in children with autism spectrum disorders (ASD) using a reticulated meta-analysis system. The results showed that physical activity (PA) led to significant improvements in all three dimensions of executive functioning in children with ASD. In particular, improvements in cognitive flexibility and inhibitory control both reached the medium effect level and were superior to improvements in working memory, which did not yet reach the medium effect level. The study also revealed differences in the effects of various exercise interventions on specific executive functions.

## Data Availability

The raw data supporting the conclusions of this article will be made available by the authors, without undue reservation.
