# Supplementary material for: Effects of different exercise interventions on executive function in children with autism spectrum disorder: a network meta-analysis
Source: Front Psychiatry. 2024 Sep 13;15:1440123. doi: 10.3389/fpsyt.2024.1440123 (PMC11427388; doi:10.3389/fpsyt.2024.1440123)
Supplement: Supplementary file 2 [file DataSheet2.docx]

Appendix 2

**Search strategy**

**S1 Cognitive Flexibility**

Figure 1 Convergent Diagnostics of Cognitive Flexibility

Figure 2 Trajectory and Density Plots of Various Interventions on Dimensions of Cognitive Flexibility

Figure 3 Consistency Model and Inconsistency Model of Cognitive Flexibility

Figure 4 Cumulative Ranking Plot of Intervention Measures on Dimensions of Cognitive Flexibility

Figure 5 Funnel Plot of Cognitive Flexibility Dimensions

Figure 6 Meta-Regression Results of Cognitive Flexibility

**S2 Inhibition Control**

Figure 7 Convergent Diagnostics of Inhibition Control

Figure 8 Trajectory and Density Plots of Various Interventions on Dimensions of Inhibition Control

Figure 9 Consistency Model and Inconsistency Model of Inhibition Control

Figure 10 Cumulative Ranking Plot of Intervention Measures on Dimensions of Inhibition Control

Figure 11 Funnel Plot of Inhibition Control Dimensions

Figure 12 Meta-Regression Results of Inhibition Control

**S3 Working Memory**

Figure 13 Convergent Diagnostics of Working Memory

Figure 14 Trajectory and Density Plots of Various Interventions on Dimensions of Working Memory

Figure 15 Consistency Model and Inconsistency Model of Working Memory

Figure 16 Cumulative Ranking Plot of Intervention Measures on Dimensions of Working Memory

Figure 17 Funnel Plot of Working Memory Dimensions

Figure 18 Meta-Regression Results of Working Memory

**Search strategy**

PubMed 1128

#1：((((Autism Spectrum Disorder [MeSH Terms]) OR (Autism Spectrum Disorders )) OR (Autistic Spectrum Disorder)) OR (Autistic Spectrum Disorders)) OR (Disorder, Autistic Spectrum)

#2：:((((Sports[MeSH Terms]) OR (((Sport[Title/Abstract]) OR (Athletics[Title/Abstract])) OR (Athletic[Title/Abstract]))) OR ((Exercise Therapy[MeSH Terms]) OR (((((((((((Remedial Exercise[Title/Abstract]) OR (Exercise, Remedial[Title/Abstract])) OR (Exercises, Remedial[Title/Abstract])) OR (Remedial Exercises[Title/Abstract])) OR (Therapy, Exercise[Title/Abstract])) OR (Exercise Therapies[Title/Abstract])) OR (Therapies, Exercise[Title/Abstract])) OR (Rehabilitation Exercise[Title/Abstract])) OR (Exercise, Rehabilitation[Title/Abstract])) OR (Exercises, Rehabilitation[Title/Abstract])) OR (Rehabilitation Exercises[Title/Abstract])))) OR ((Exercise[MeSH Terms]) OR ((((((((((((((((((((((((Exercises[Title/Abstract]) OR (Physical Activity[Title/Abstract])) OR (Activities, Physical[Title/Abstract])) OR (Activity, Physical[Title/Abstract])) OR (Physical Activities[Title/Abstract])) OR (Exercise, Physical[Title/Abstract])) OR (Exercises, Physical[Title/Abstract])) OR (Physical Exercise[Title/Abstract])) OR (Physical Exercises[Title/Abstract])) OR (Acute Exercise[Title/Abstract])) OR (Acute Exercises[Title/Abstract])) OR (Exercise, Acute[Title/Abstract])) OR (Exercises, Acute[Title/Abstract])) OR (Exercise, Isometric[Title/Abstract])) OR (Exercises, Isometric[Title/Abstract])) OR (Isometric Exercises[Title/Abstract])) OR (Isometric Exercise[Title/Abstract])) OR (Exercise, Aerobic[Title/Abstract])) OR (Exercise, Aerobic[Title/Abstract])) OR (Aerobic Exercise[Title/Abstract])) OR (Aerobic Exercises[Title/Abstract])) OR (Exercises, Aerobic[Title/Abstract])) OR (Exercise Training[Title/Abstract])) OR (Trainings, Exercise[Title/Abstract])))) OR (((((((((((((((((((((((((((((((((((((((((((Baseball[Title/Abstract]) OR (Basketball[Title/Abstract])) OR (Bicycling[Title/Abstract])) OR (Boxing[Title/Abstract])) OR (Cricket Sport[Title/Abstract])) OR (Football[Title/Abstract])) OR (Golf[Title/Abstract])) OR (Gymnastics[Title/Abstract])) OR (Hockey[Title/Abstract])) OR (Martial Arts[Title/Abstract])) OR (Mountaineering[Title/Abstract])) OR (Racquet[Title/Abstract])) OR (Return[Title/Abstract])) OR (Sport[Title/Abstract])) OR (Sports[Title/Abstract])) OR (Rugby[Title/Abstract])) OR (Running[Title/Abstract])) OR (Skating[Title/Abstract])) OR (Snow Sports[Title/Abstract])) OR (Soccer[Title/Abstract])) OR (train[Title/Abstract])) OR (fitness[Title/Abstract])) OR (aerobic[Title/Abstract])) OR (walking[Title/Abstract])) OR (high intensity interval[Title/Abstract])) OR (resistance[Title/Abstract])) OR (core stability[Title/Abstract])) OR (dance[Title/Abstract])) OR (breathing exercise[Title/Abstract])) OR (virtual reality exercise[Title/Abstract])) OR (whole body vibration exercise[Title/Abstract])) OR (stretching[Title/Abstract])) OR (body ⁃ mind exercise[Title/Abstract])) OR (Yoga[Title/Abstract])) OR (pilates[Title/Abstract])) OR (Tai Chi[Title/Abstract])) OR (Taijiquan[Title/Abstract])) OR (Health Qigong[Title/Abstract])) OR (Yijinjing[Title/Abstract])) OR (Wuqinxi[Title/Abstract])) OR (Liuzijue[Title/Abstract])) OR (Baduanjin[Title/Abstract])) OR (multicomponent exercise[Title/Abstract]))

#3：((((((((((((((((((((((((Child[MeSH Terms]) OR (Adolescent[MeSH Terms])) OR (Young Adult[MeSH Terms])) OR (Minor[MeSH Terms])) OR (Adolescen[Title/Abstract])) OR (Teen[Title/Abstract])) OR (Teenager[Title/Abstract])) OR (Youth[Title/Abstract])) OR (Minors[Title/Abstract])) OR (Minor[Title/Abstract])) OR (Child[Title/Abstract])) OR (Child[Title/Abstract])) OR (Kid[Title/Abstract])) OR (Kids[Title/Abstract])) OR (Girl[Title/Abstract])) OR (Boy[Title/Abstract])) OR (Under age[Title/Abstract])) OR (Underage[Title/Abstract])) OR (Young people[Title/Abstract])) OR (young person[Title/Abstract])) OR (Pubescen[Title/Abstract])) OR (Young Adult[Title/Abstract])) OR (School age[Title/Abstract])) OR (Preschool[Title/Abstract])) OR (Student[Title/Abstract])

#4：#1 AND #2 AND #3

WOS 6753

#1：((((TS=(Autism Spectrum Disorder)) OR TS=(Autism Spectrum Disorders)) OR TS=(Autistic Spectrum Disorder)) OR TS=(Autistic Spectrum Disorders)) OR TS=(Disorder, Autistic Spectrum)

#2：(TS=(Equine-Assisted Therapy)) OR (TS=(Animal Assisted Therapy)) OR (TS=(Baseball)) OR (TS=(Basketball)) OR (TS=(Bicycling)) OR (TS=(Boxing)) OR (TS=(Cricket Sport)) OR (TS=(Football)) OR (TS=(Golf)) OR (TS=(Gymnastics)) OR (TS=(Hockey)) OR (TS=(Martial Arts)) OR (TS=(Mountaineering)) OR (TS=(Racquet Sports)) OR (TS=(Return to Sport)) OR (TS=(Rugby)) OR (TS=(Running)) OR (TS=(Skating)) OR (TS=(Snow Sports)) OR (TS=(Soccer)) OR (TS=(train)) OR (TS=(fitness)) OR (TS=(aerobic)) OR (TS=(walking)) OR (TS=(high intensity interval)) OR (TS=(resistance)) OR (TS=(core stability)) OR (TS=(dance)) OR (TS=(breathing exercise)) OR (TS=(virtual reality exercise)) OR (TS=(whole body vibration exercise)) OR (TS=(stretching)) OR (TS=(body ⁃ mind exercise)) OR (TS=(Yoga)) OR (TS=(pilates)) OR (TS=(Tai Chi)) OR (TS=(Taijiquan)) OR (TS=(Health Qigong)) OR (TS=(Yijinjing)) OR (TS=(Wuqinxi)) OR (TS=(Liuzijue)) OR (TS=(Baduanjin)) OR (TS=(multicomponent exercise)) OR (((TS=(Sports)) OR TS=(Sport)) OR TS=(Athletics)) OR TS=(Athletic)OR (((((((((((TS=(Exercise Therapy)) OR TS=(Remedial Exercise)) OR TS=(Exercise, Remedial)) OR TS=(Exercises, Remedial)) OR TS=(Remedial Exercises)) OR TS=(Therapy, Exercise)) OR TS=(Exercise Therapies)) OR TS=(Therapies, Exercise)) OR TS=(Rehabilitation Exercise)) OR TS=(Exercise, Rehabilitation)) OR TS=(Exercises, Rehabilitation)) OR TS=(Rehabilitation Exercises)

OR (((((((((((((((((((((((((TS=(Exercise)) OR TS=(Exercises)) OR TS=(Physical Activity)) OR TS=(Activities, Physical)) OR TS=(Activity, Physical)) OR TS=(Physical Activities)) OR TS=(Exercise, Physical)) OR TS=(Exercises, Physical)) OR TS=(Physical Exercise)) OR TS=(Physical Exercises)) OR TS=( Acute Exercise)) OR TS=(Acute Exercises)) OR TS=(Exercise, Acute)) OR TS=(Exercises, Acute)) OR TS=(Exercise, Isometric)) OR TS=(Exercises, Isometric)) OR TS=(Isometric Exercises)) OR TS=(Isometric Exercise)) OR TS=(Exercise, Aerobic)) OR TS=(Aerobic Exercise)) OR TS=(Aerobic Exercises)) OR TS=(Exercises, Aerobic)) OR TS=(Exercise Training)) OR TS=(Exercise Trainings)) OR TS=(Training, Exercise)) OR TS=(Trainings, Exercise)

#3：((((((((((((((((((((((((TS=(Child)) OR TS=(Adolescent)) OR TS=(Young Adult )) OR TS=(Minor )) OR TS=(Adolescen)) OR TS=(Teen)) OR TS=(Teenager)) OR TS=(Youth)) OR TS=(Minors)) OR TS=(Minor)) OR TS=(Child)) OR TS=(Kid)) OR TS=(Kids)) OR TS=(Girl)) OR TS=(Boy)) OR TS=(Under age)) OR TS=(Underage)) OR TS=(Young people )) OR TS=(young person )) OR TS=(Prepubescen)) OR TS=(Pubescen)) OR TS=(Young Adult)) OR TS=(School age)) OR TS=(Preschool)) OR TS=(Student)

#4：#1 AND #2 AND #3

Cochrane 413

#1：Autism Spectrum Disorder

#2：(Autism Spectrum Disorders ):ab,ti,kw OR (Autistic Spectrum Disorder ):ab,ti,kw OR (Autistic Spectrum Disorders ):ab,ti,kw OR (Disorder, Autistic Spectrum ):ab,ti,kw

#3：Therapeutics

#4：(Therapeutic ):ab,ti,kw OR (Therapy ):ab,ti,kw OR (Therapies ):ab,ti,kw OR (Treatment ):ab,ti,kw OR (Treatments ):ab,ti,kw

#5：Sports

#6：(Sport ):ab,ti,kw OR (Athletics ):ab,ti,kw OR (Athletic ):ab,ti,kw

#7：Exercise

#8：(Exercises ):ab,ti,kw OR (Physical Activity ):ab,ti,kw OR (Activities, Physical ):ab,ti,kw OR (Activity, Physical ):ab,ti,kw OR (Physical Activities ):ab,ti,kw OR (Exercise, Physical ):ab,ti,kw OR (Exercises, Physical ):ab,ti,kw OR (Physical Exercise ):ab,ti,kw OR (Physical Exercises ):ab,ti,kw OR (Acute Exercise ):ab,ti,kw OR (Acute Exercises ):ab,ti,kw OR (Exercise, Acute ):ab,ti,kw OR (Exercises, Acute ):ab,ti,kw OR (Exercise, Isometric ):ab,ti,kw OR (Exercises, Isometric ):ab,ti,kw OR (Isometric Exercises ):ab,ti,kw OR (Isometric Exercise ):ab,ti,kw OR (Exercise, Aerobic ):ab,ti,kw OR (Aerobic Exercise ):ab,ti,kw OR (Aerobic Exercises ):ab,ti,kw OR (Exercises, Aerobic ):ab,ti,kw OR (Exercise Training ):ab,ti,kw OR (Exercise Trainings ):ab,ti,kw OR (Training, Exercise ):ab,ti,kw OR (Trainings, Exercise ):ab,ti,kw

#9：(Baseball ):ab,ti,kw OR (Basketball ):ab,ti,kw OR (Bicycling ):ab,ti,kw OR (Boxing ):ab,ti,kw OR (Cricket Sport ):ab,ti,kw OR (Football ):ab,ti,kw OR (Golf ):ab,ti,kw OR (Gymnastics ):ab,ti,kw OR (Hockey ):ab,ti,kw OR (Martial Arts ):ab,ti,kw OR (Mountaineering ):ab,ti,kw OR (Racquet Sports ):ab,ti,kw OR (Return to Sport ):ab,ti,kw OR (Rugby ):ab,ti,kw OR (Running ):ab,ti,kw OR (Skating ):ab,ti,kw OR (Snow Sports ):ab,ti,kw OR (Soccer ):ab,ti,kw OR (train ):ab,ti,kw OR (fitness ):ab,ti,kw OR (aerobic ):ab,ti,kw OR (walking ):ab,ti,kw OR (high intensity interval ):ab,ti,kw OR (resistance ):ab,ti,kw OR (core stability ):ab,ti,kw OR (dance ):ab,ti,kw OR (breathing exercise ):ab,ti,kw OR (virtual reality exercise ):ab,ti,kw OR (whole body vibration exercise ):ab,ti,kw OR (stretching ):ab,ti,kw OR (body mind exercise ):ab,ti,kw OR (Yoga ):ab,ti,kw OR (pilates ):ab,ti,kw OR (Tai Chi ):ab,ti,kw OR (Taijiquan ):ab,ti,kw OR (Health Qigong ):ab,ti,kw OR (Yijinjing ):ab,ti,kw OR (Wuqinxi ):ab,ti,kw OR (Liuzijue ):ab,ti,kw OR (Baduanjin ):ab,ti,kw OR (multicomponent exercise ):ab,ti,kw OR (Equine-Assisted Therapy ):ab,ti,kw OR (Animal Assisted Therapy ):ab,ti,kw

#10：Child OR Adolescent OR Young Adult OR Minor

#11：(Children ):ab,ti,kw OR (Children ):ab,ti,kw OR (Child ):ab,ti,kw OR (Adolescent ):ab,ti,kw OR (Young Adult ):ab,ti,kw OR (Minor ):ab,ti,kw OR (Adolescen ):ab,ti,kw OR (Teen ):ab,ti,kw OR (Teenager ):ab,ti,kw OR (Youth ):ab,ti,kw OR (Minors ):ab,ti,kw OR (Minor ):ab,ti,kw OR (Child ):ab,ti,kw OR (Kid ):ab,ti,kw OR (Girl ):ab,ti,kw OR (Boy ):ab,ti,kw OR (Under age ):ab,ti,kw OR (Underage ):ab,ti,kw OR (Young people):ab,ti,kw OR (young person ):ab,ti,kw OR (Prepubescen ):ab,ti,kw OR (Pubescen ):ab,ti,kw OR (Young Adult ):ab,ti,kw OR (School age ):ab,ti,kw OR (Preschool ):ab,ti,kw OR (Student ):ab,ti,kw

#12：#1 OR #2

#13：#3 OR #4 #5 OR #6 #7 OR #8 OR #9

#14：#10 OR #11

#15：#12 AND #13 AND #14

Embase 1422

#1：autism AND spectrum AND disorder

#2：'autism spectrum disorders':ab,ti OR 'autistic spectrum disorder':ab,ti OR 'autistic spectrum disorders':ab,ti OR 'disorder, autistic spectrum':ab,ti

#3：sports

#4：'sport':ab,ti OR 'athletics':ab,ti OR 'athletic':ab,ti

#5：exercise AND therapy

#6：'remedial exercise':ab,ti OR 'exercise, remedial':ab,ti OR 'exercises, remedial':ab,ti OR 'remedial exercises':ab,ti OR 'therapy, exercise':ab,ti OR 'exercise therapies':ab,ti OR 'therapies, exercise':ab,ti OR 'rehabilitation exercise':ab,ti OR 'exercise, rehabilitation':ab,ti OR 'exercises, rehabilitation':ab,ti OR 'rehabilitation exercises':ab,ti

#7：exercise

#8：'exercises':ab,ti OR 'physical activity':ab,ti OR 'activities, physical':ab,ti OR 'activity, physical':ab,ti OR 'physical activities':ab,ti OR 'exercise, physical':ab,ti OR 'exercises, physical':ab,ti OR 'physical exercise':ab,ti OR 'physical exercises':ab,ti OR 'acute exercise':ab,ti OR 'acute exercises':ab,ti OR 'exercise, acute':ab,ti OR 'exercises, acute':ab,ti OR 'exercise, isometric':ab,ti OR 'exercises, isometric':ab,ti OR 'isometric exercises':ab,ti OR 'isometric exercise':ab,ti OR 'exercise, aerobic':ab,ti OR 'aerobic exercise':ab,ti OR 'aerobic exercises':ab,ti OR 'exercises, aerobic':ab,ti OR 'exercise training':ab,ti OR 'exercise trainings':ab,ti OR 'training, exercise':ab,ti OR 'trainings, exercise':ab,ti

#9：'baseball':ab,ti OR 'basketball':ab,ti OR 'bicycling':ab,ti OR 'boxing':ab,ti OR 'cricket sport':ab,ti OR 'football':ab,ti OR 'golf':ab,ti OR 'gymnastics':ab,ti OR 'hockey':ab,ti OR 'martial arts':ab,ti OR 'mountaineering':ab,ti OR 'racquet sports':ab,ti OR 'return to sport':ab,ti OR 'rugby':ab,ti OR 'running':ab,ti OR 'skating':ab,ti OR 'snow sports':ab,ti OR 'soccer':ab,ti OR 'train':ab,ti OR 'fitness':ab,ti OR 'aerobic':ab,ti OR 'walking':ab,ti OR 'high intensity interval':ab,ti OR 'resistance':ab,ti OR 'core stability':ab,ti OR 'dance':ab,ti OR 'breathing exercise':ab,ti OR 'virtual reality exercise':ab,ti OR 'whole body vibration exercise':ab,ti OR 'stretching':ab,ti OR 'body mind exercise':ab,ti OR 'yoga':ab,ti OR 'pilates':ab,ti OR 'tai chi':ab,ti OR 'taijiquan':ab,ti OR 'health qigong':ab,ti OR 'yijinjing':ab,ti OR 'wuqinxi':ab,ti OR 'liuzijue':ab,ti OR 'baduanjin':ab,ti OR 'multicomponent exercise':ab,ti OR 'equine-assisted therapy':ab,ti OR 'animal assisted therapy':ab,ti

#10：'child'/exp OR child

#11：'children':ab,ti OR 'adolescent':ab,ti OR 'adolescen':ab,ti OR 'teen':ab,ti OR 'teenager':ab,ti OR 'youth':ab,ti OR 'minors':ab,ti OR 'minor':ab,ti OR 'child':ab,ti OR 'kid':ab,ti OR 'girl':ab,ti OR 'boy':ab,ti OR 'under age':ab,ti OR 'underage':ab,ti OR 'young people':ab,ti OR 'young person':ab,ti OR 'prepubescen':ab,ti OR 'pubescen':ab,ti OR 'young adult':ab,ti OR 'school age':ab,ti OR 'preschool':ab,ti OR 'student':ab,ti

#12：#1 OR #2

#13：#3 OR #4 #5 OR #6 #7 OR #8 OR #9

#14：#10 OR #11

#15：#12 AND #13 AND #14

## Cognitive Flexibility

**Figure 1** Convergent Diagnostics of Cognitive Flexibility


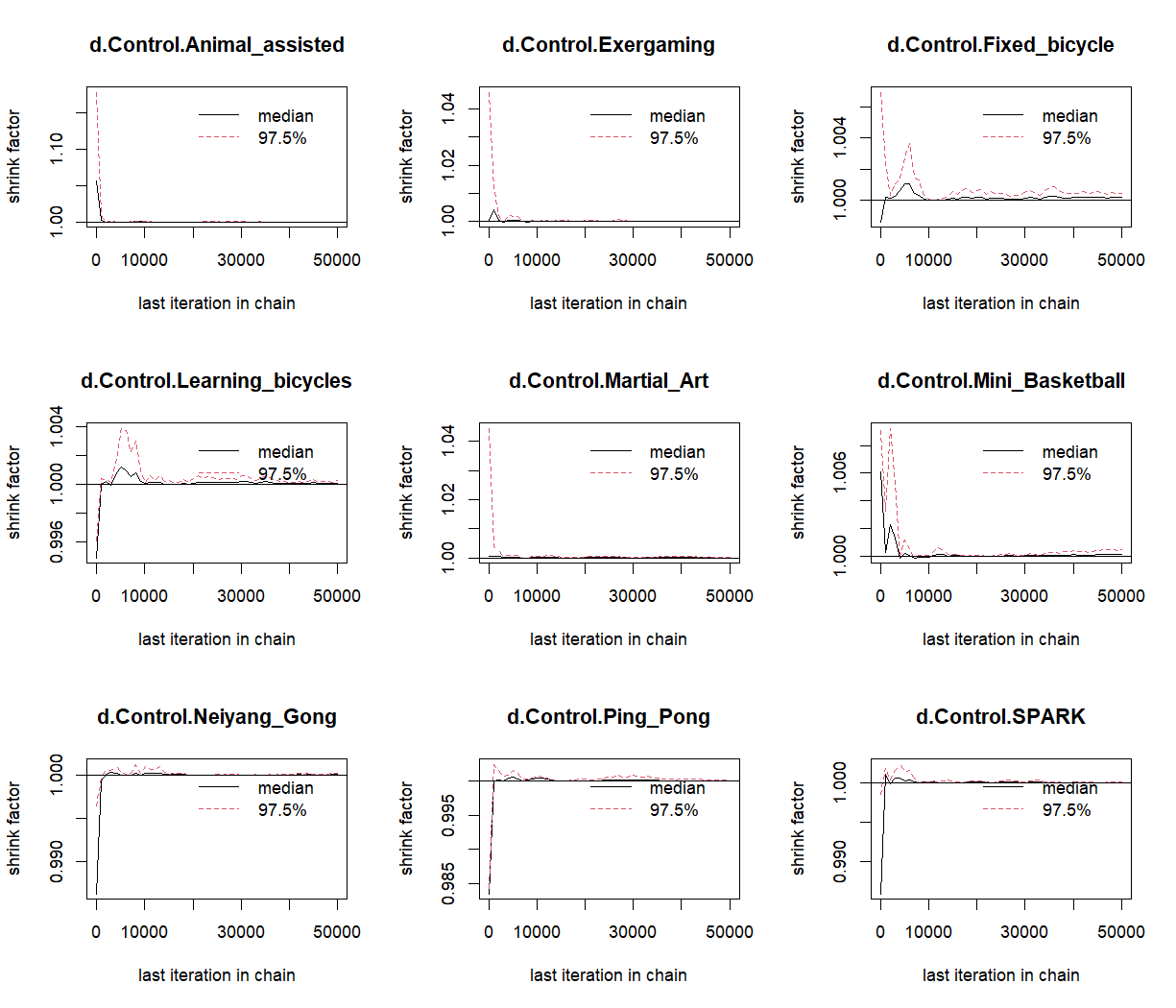


**Figure 2** Trajectory and Density Plots of Various Interventions on Dimensions of Cognitive Flexibility


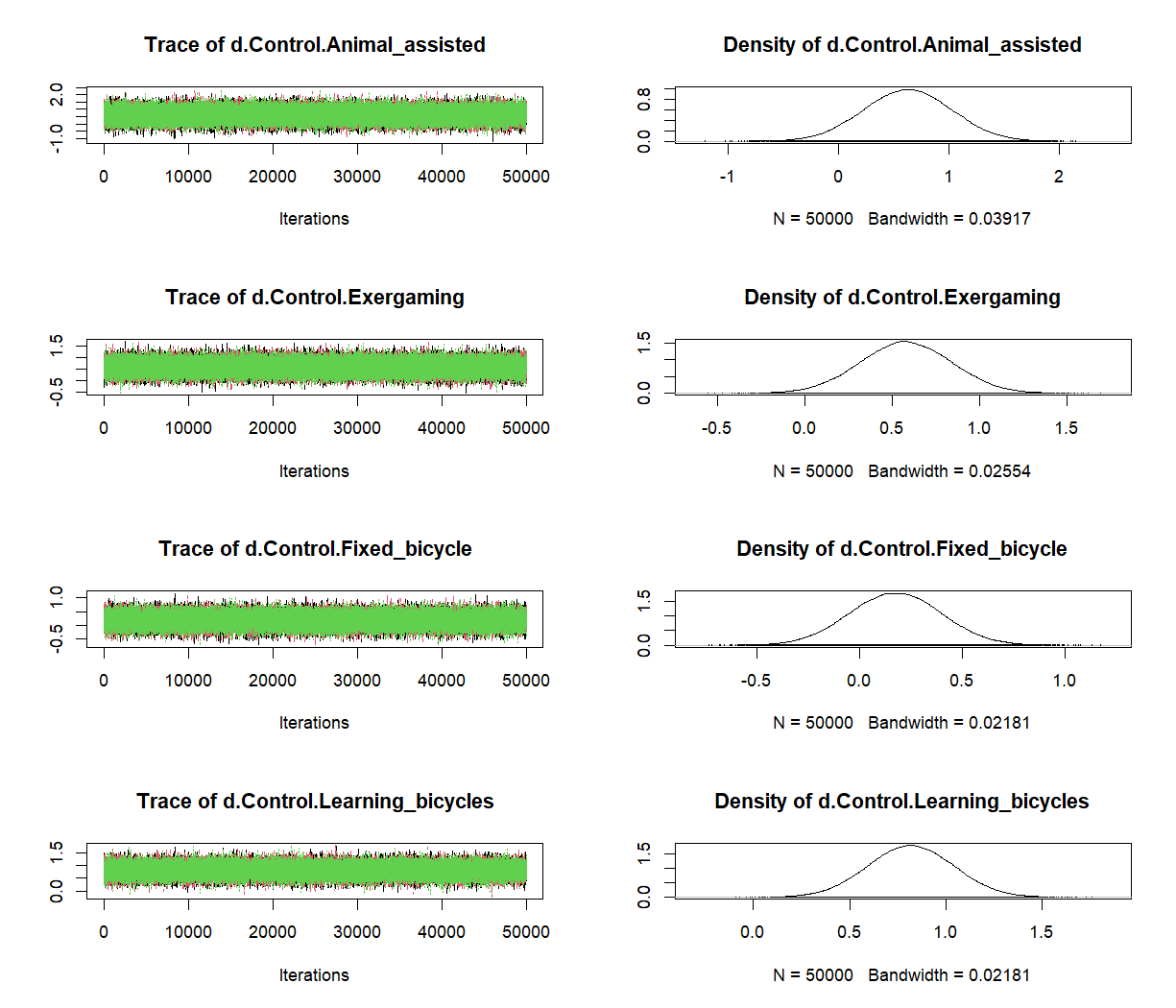


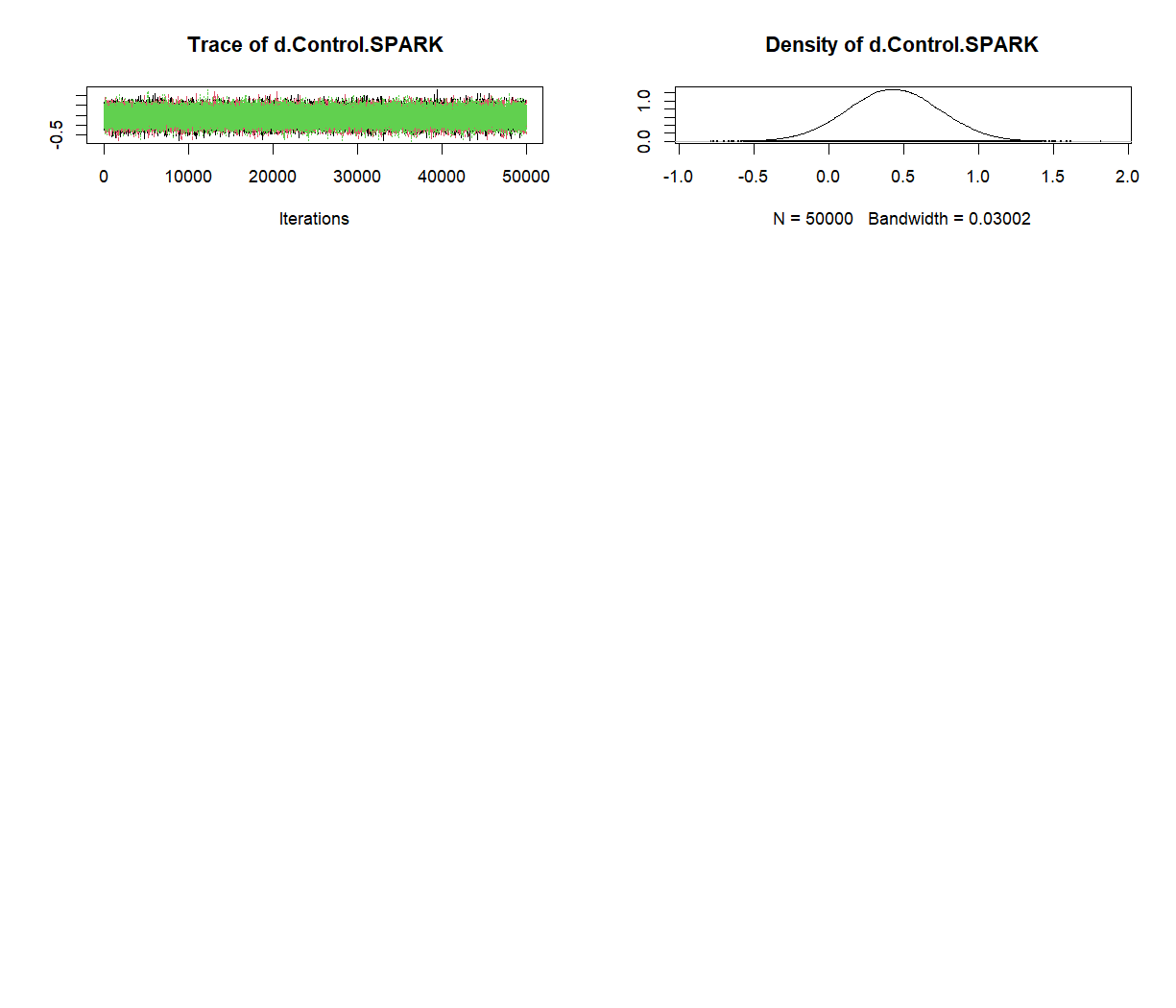


**Figure 3** Consistency Model and Inconsistency Model of Cognitive Flexibility


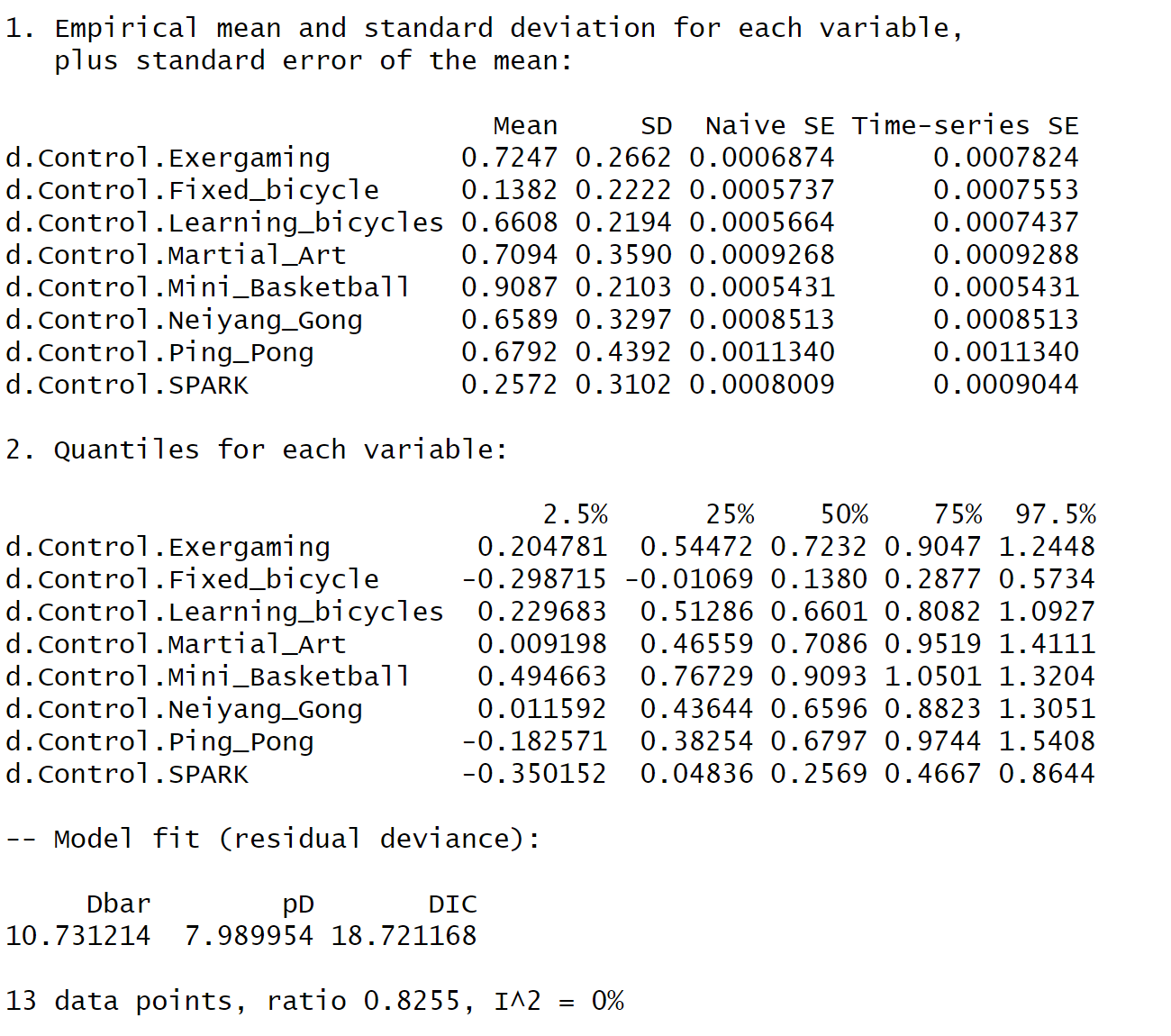


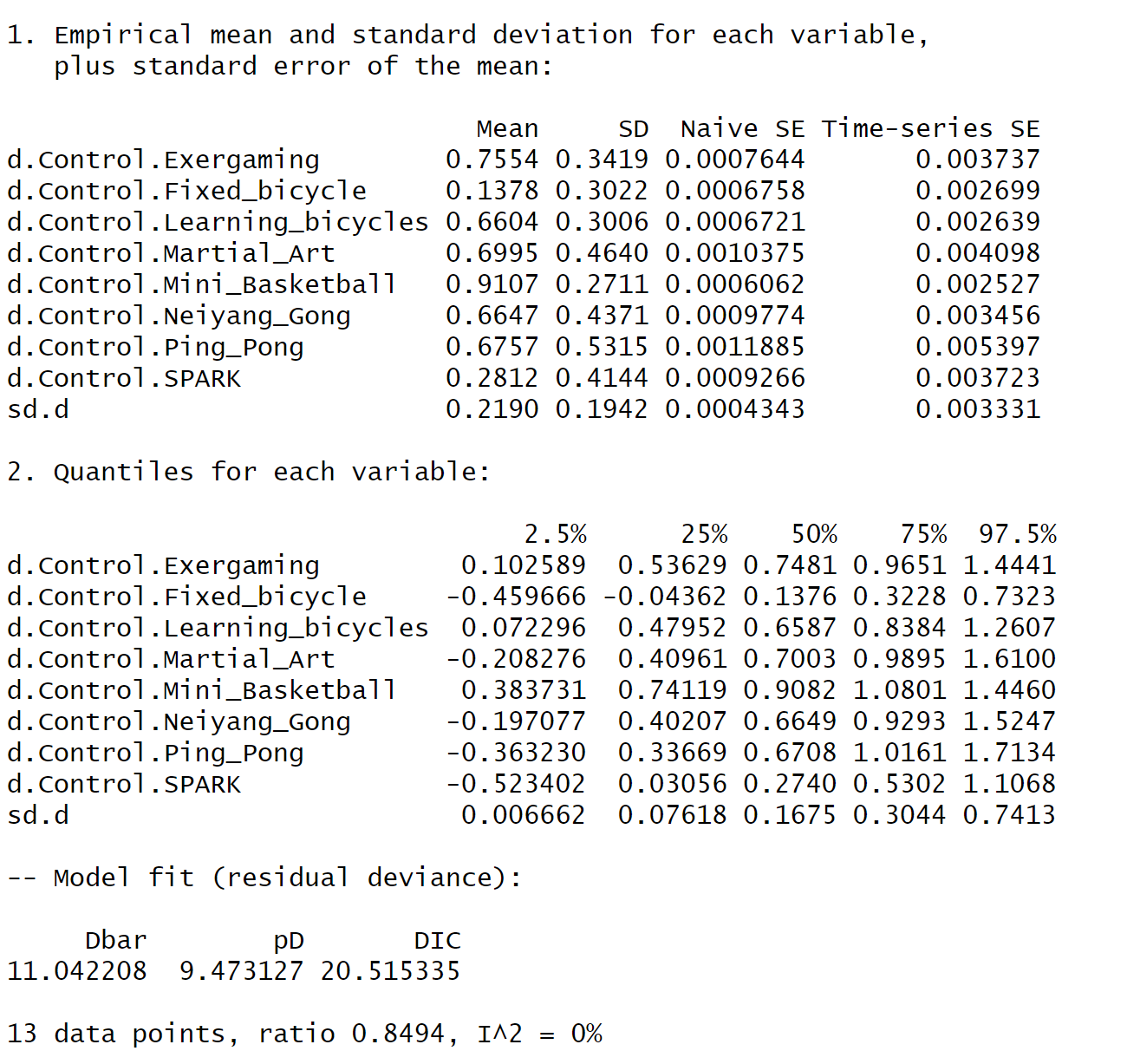


**Figure 4** Cumulative Ranking Plot of Intervention Measures on Dimensions of Cognitive Flexibility


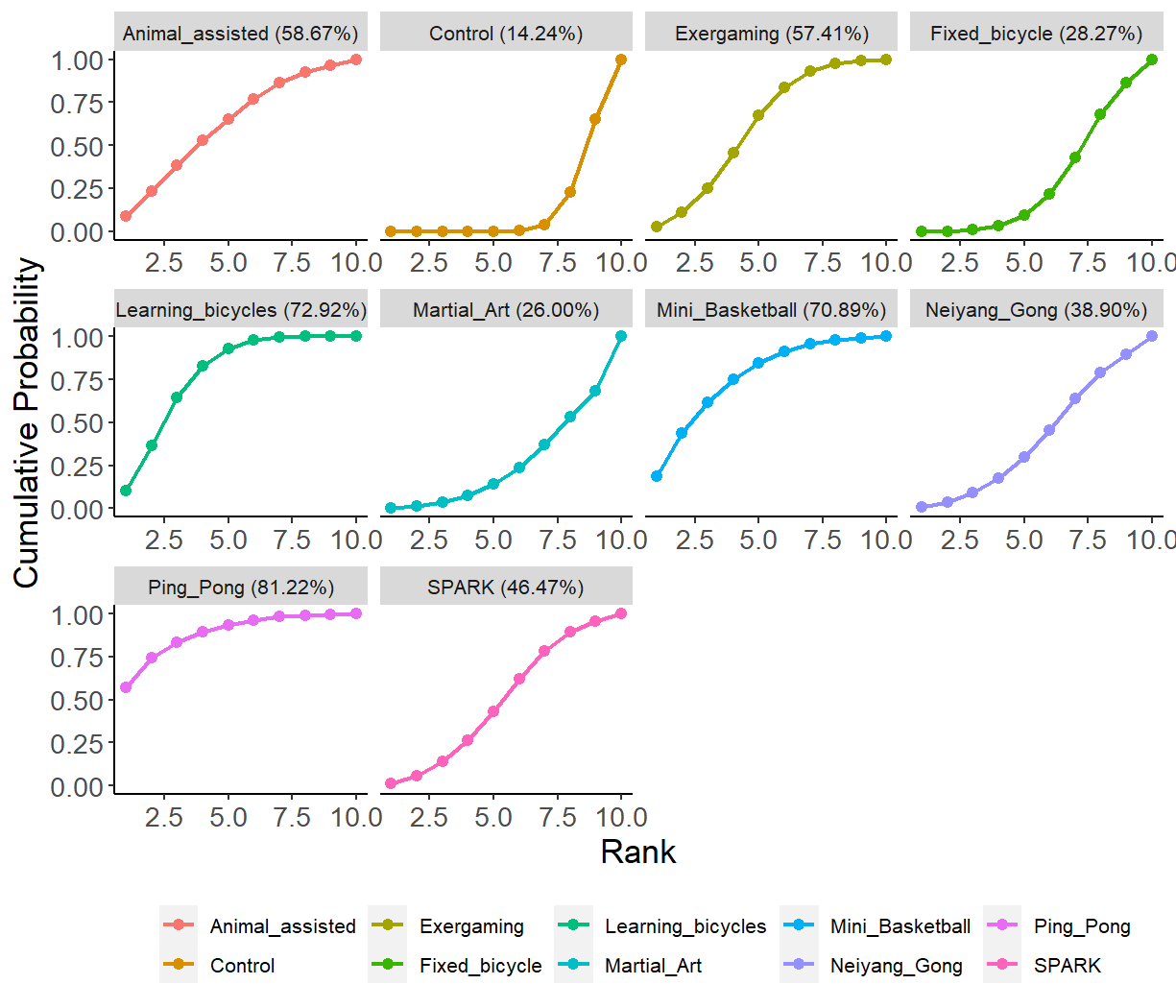


**Figure 5** Funnel Plot of Cognitive Flexibility Dimensions


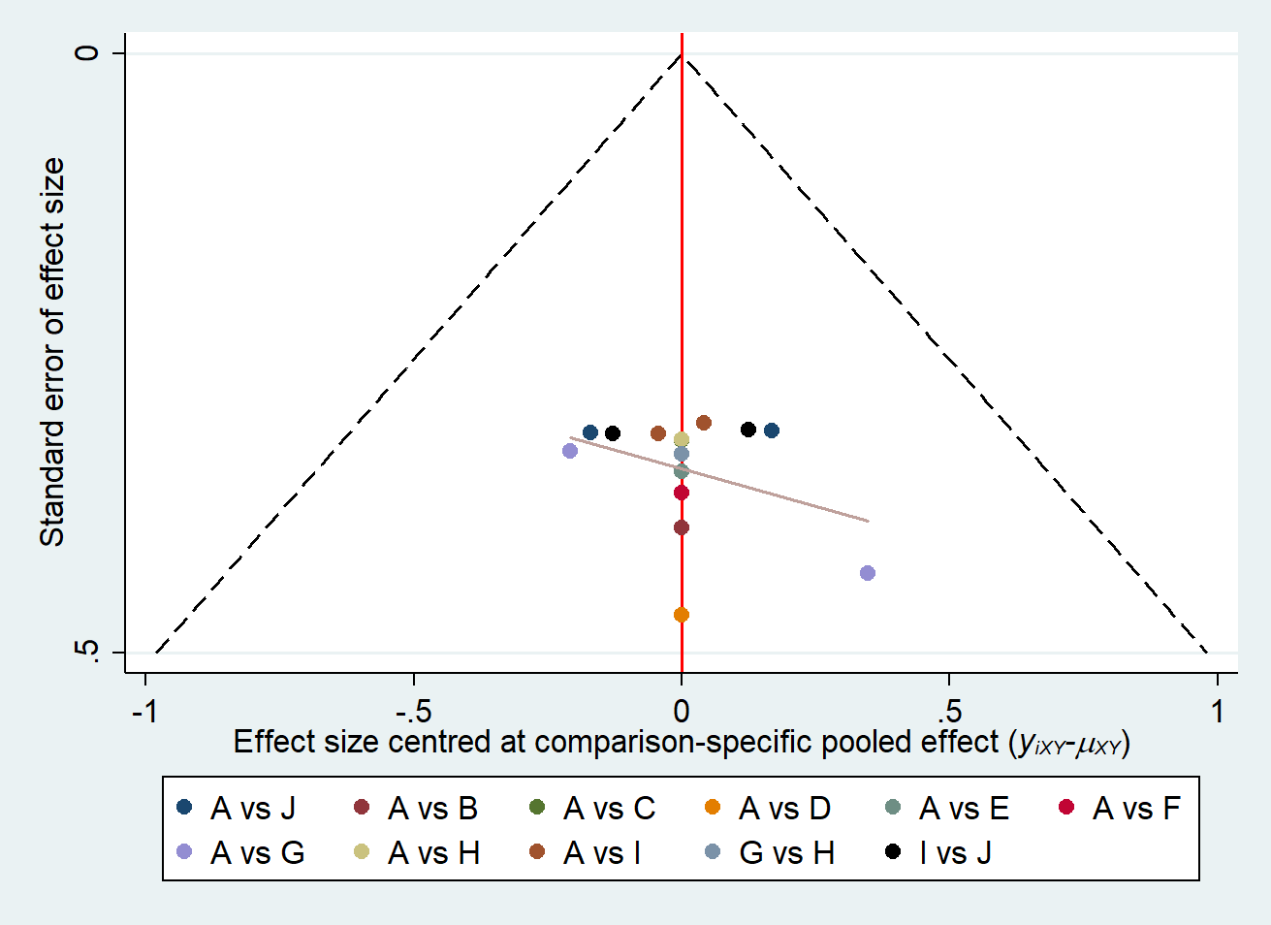


**Figure 6** Meta-Regression Results of Cognitive Flexibility


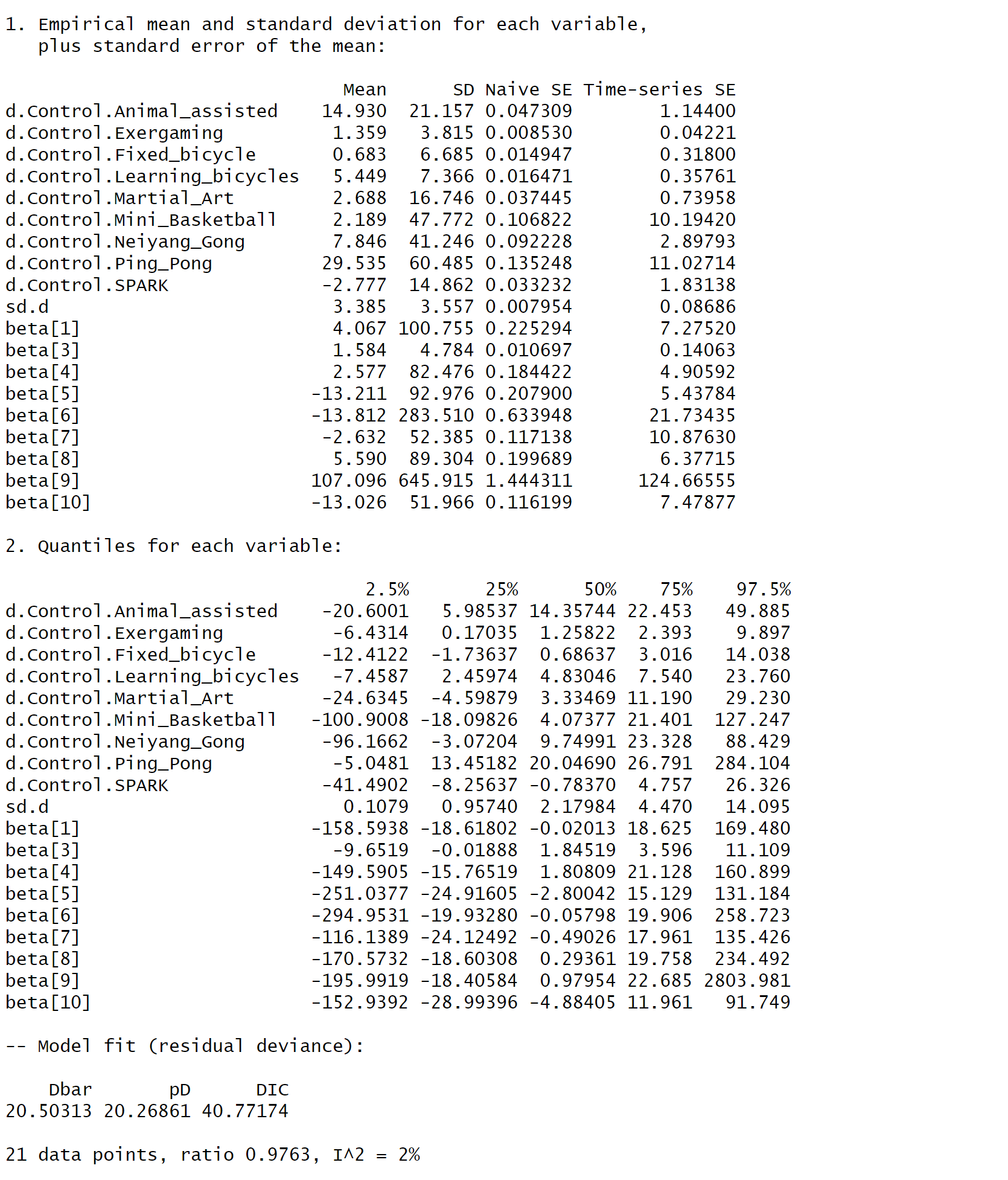

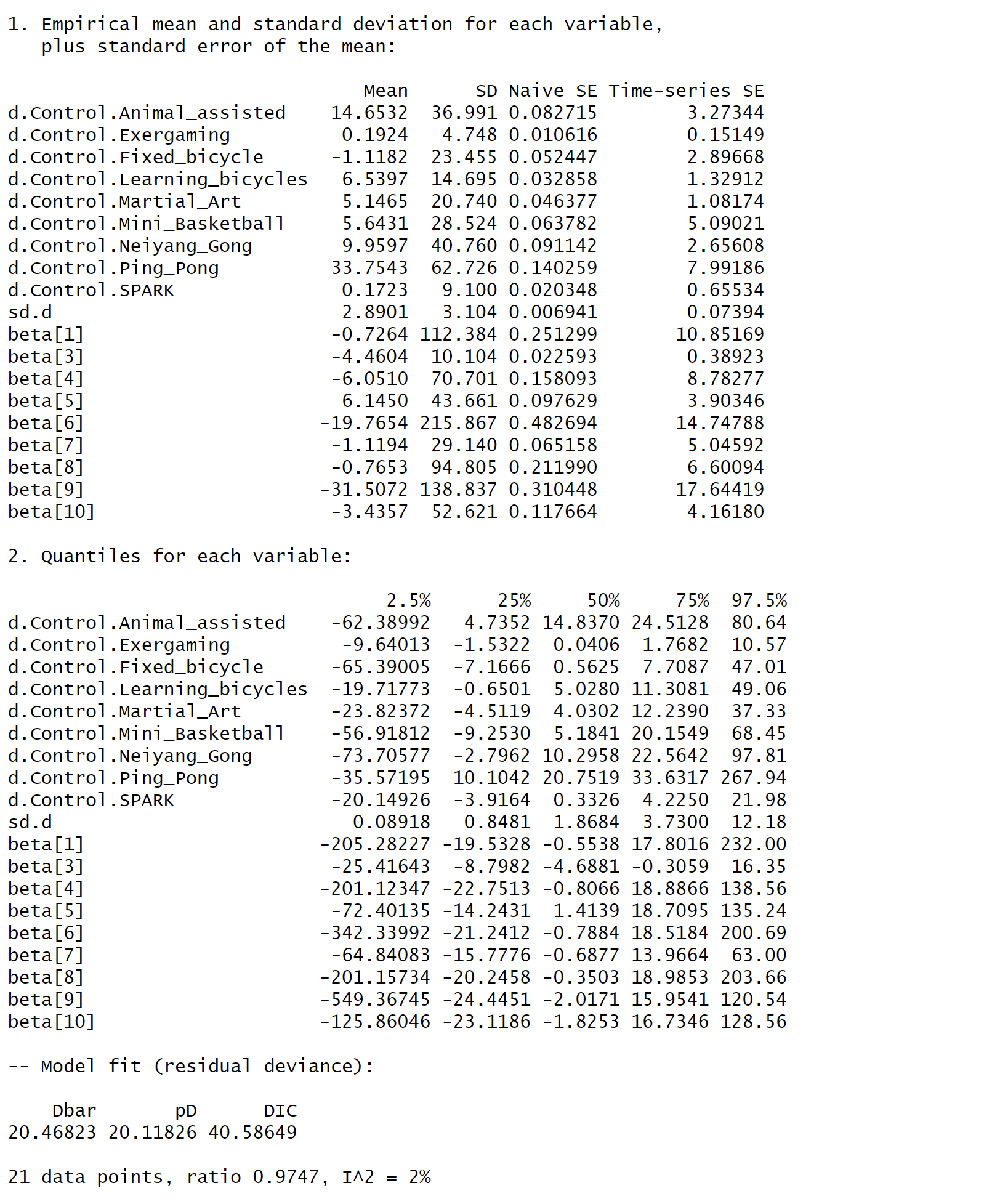


Average Age Total duration/hour


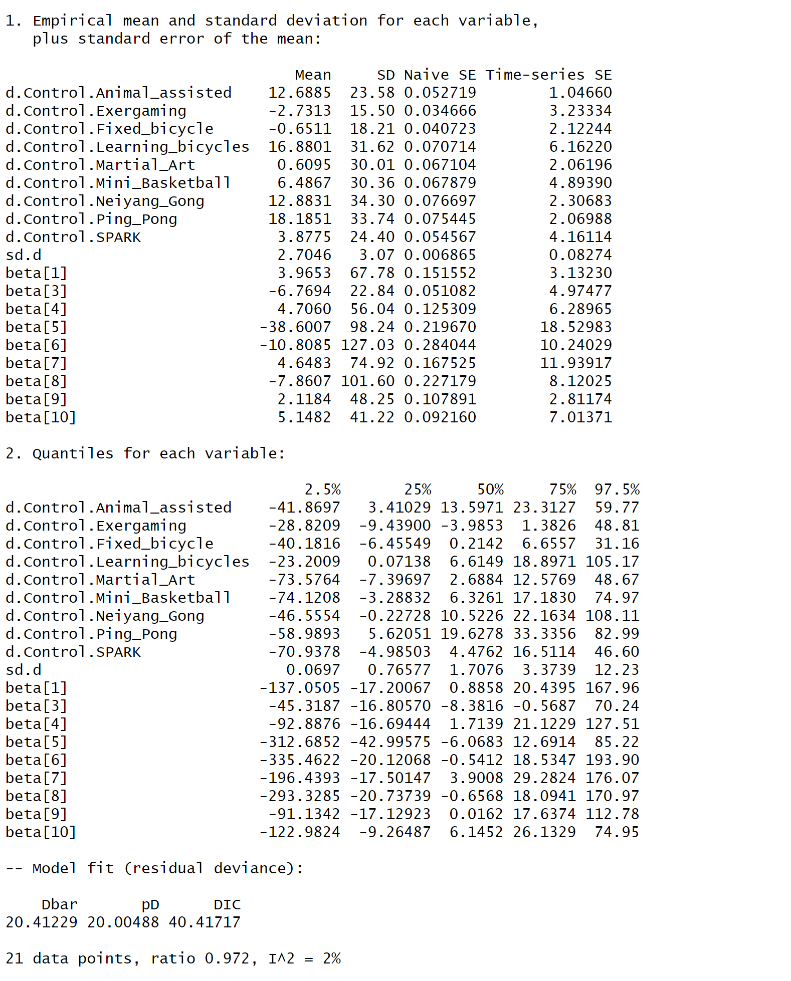

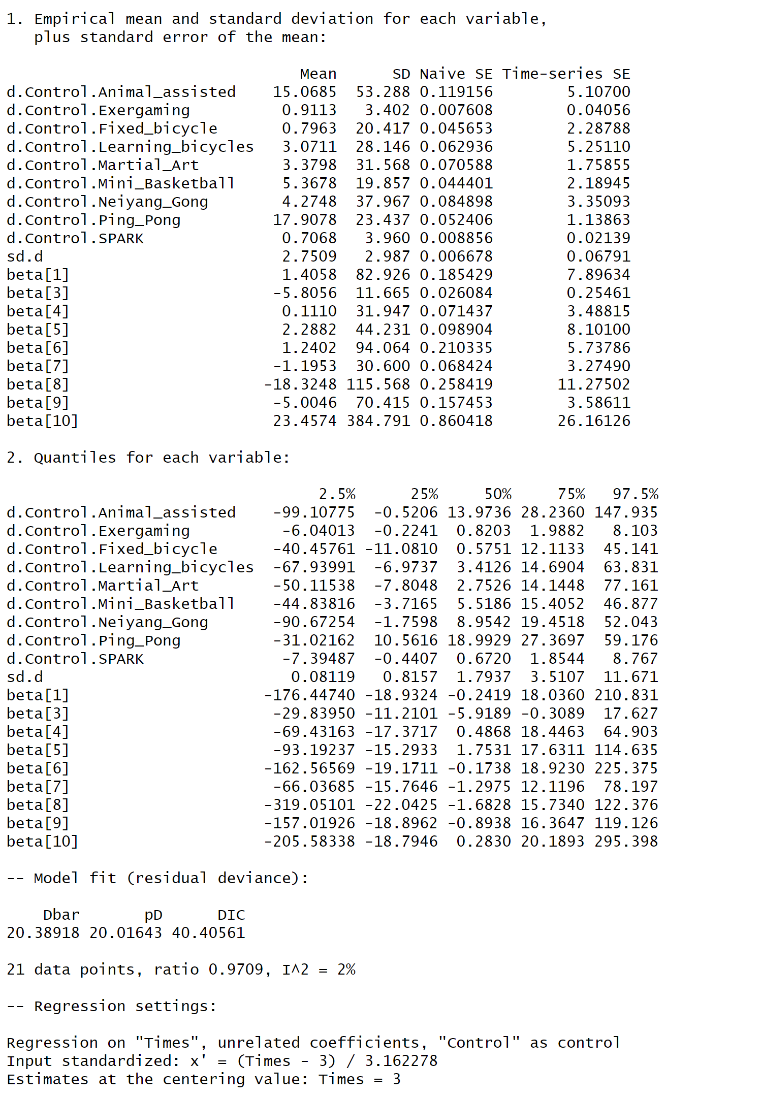


Duration per minute Weekly intervention frequency


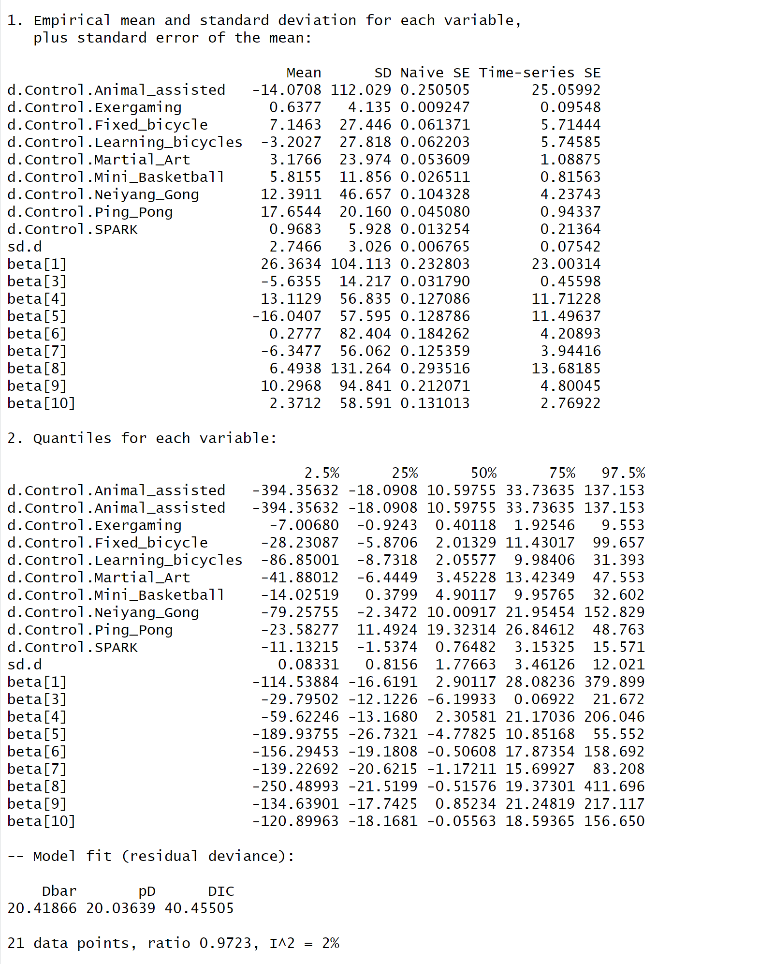

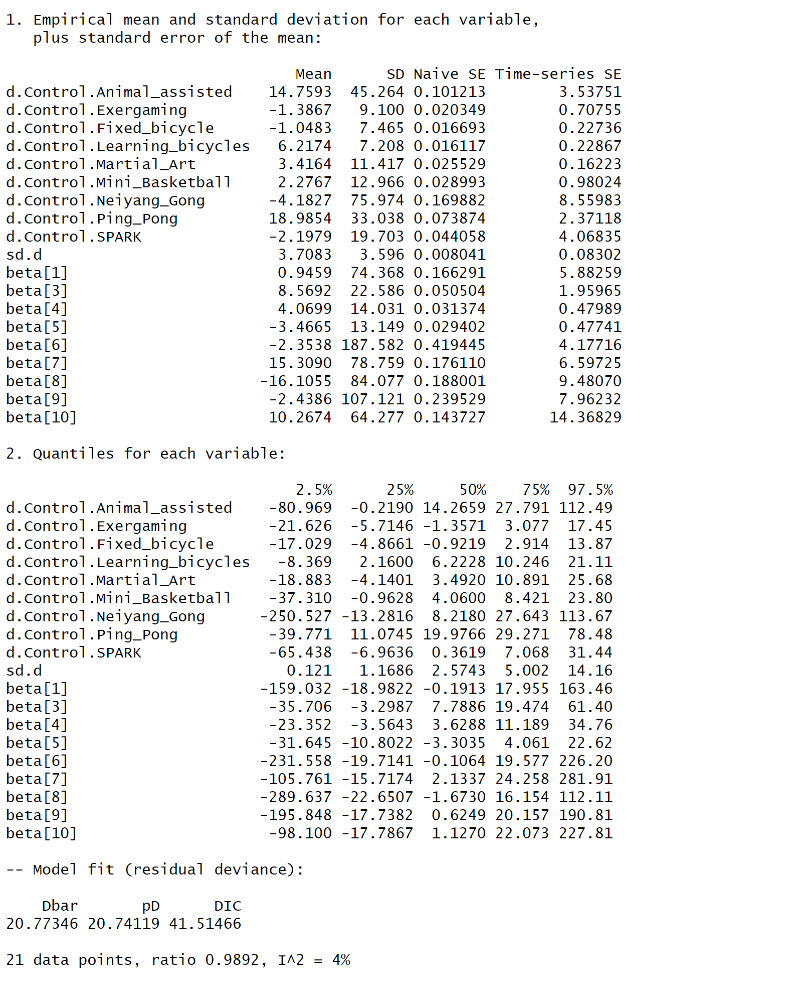


Duration Week Year of publication

## Inhibition Control

**Figure 7** Convergent Diagnostics of Inhibition Control


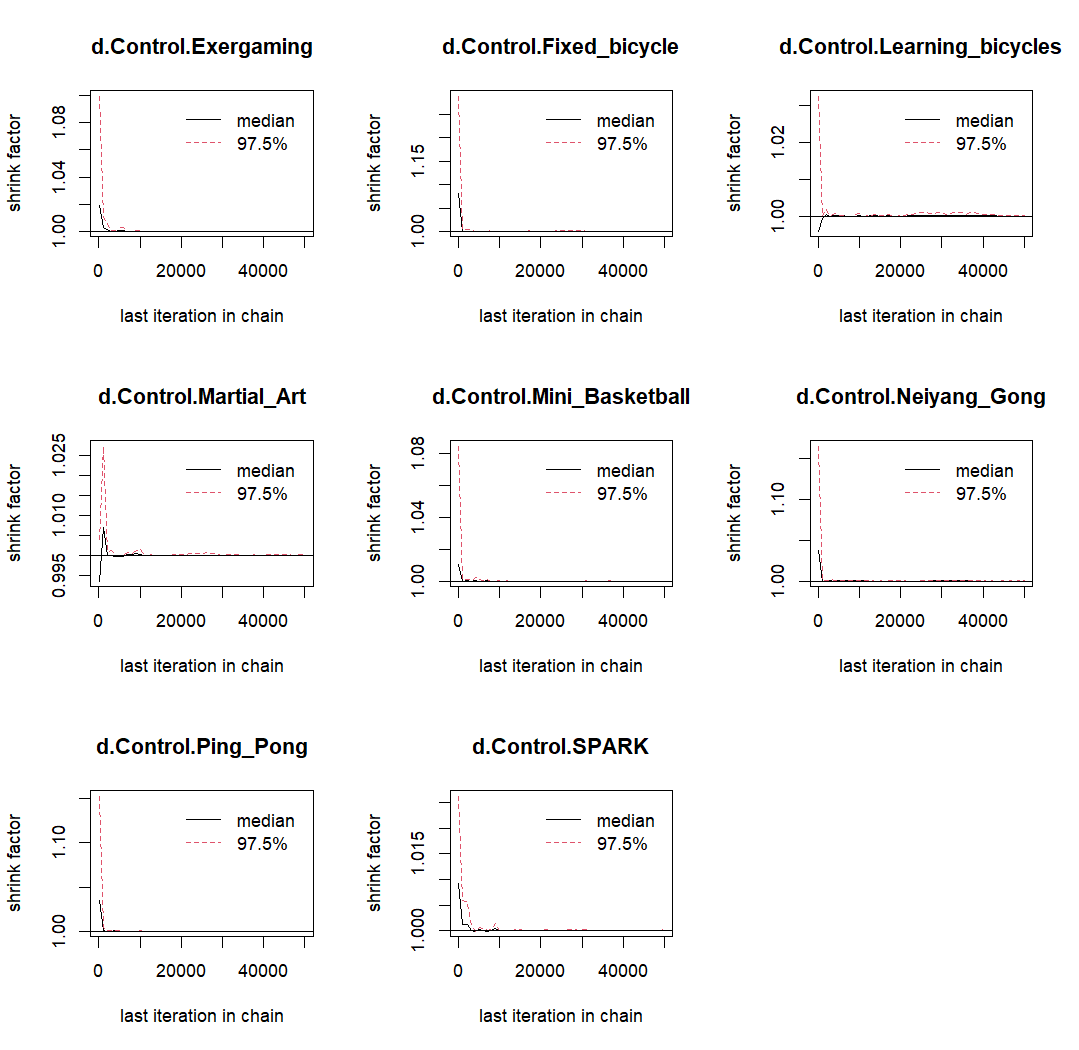


**Figure 8** Trajectory and Density Plots of Various Interventions on Dimensions of Inhibition Control


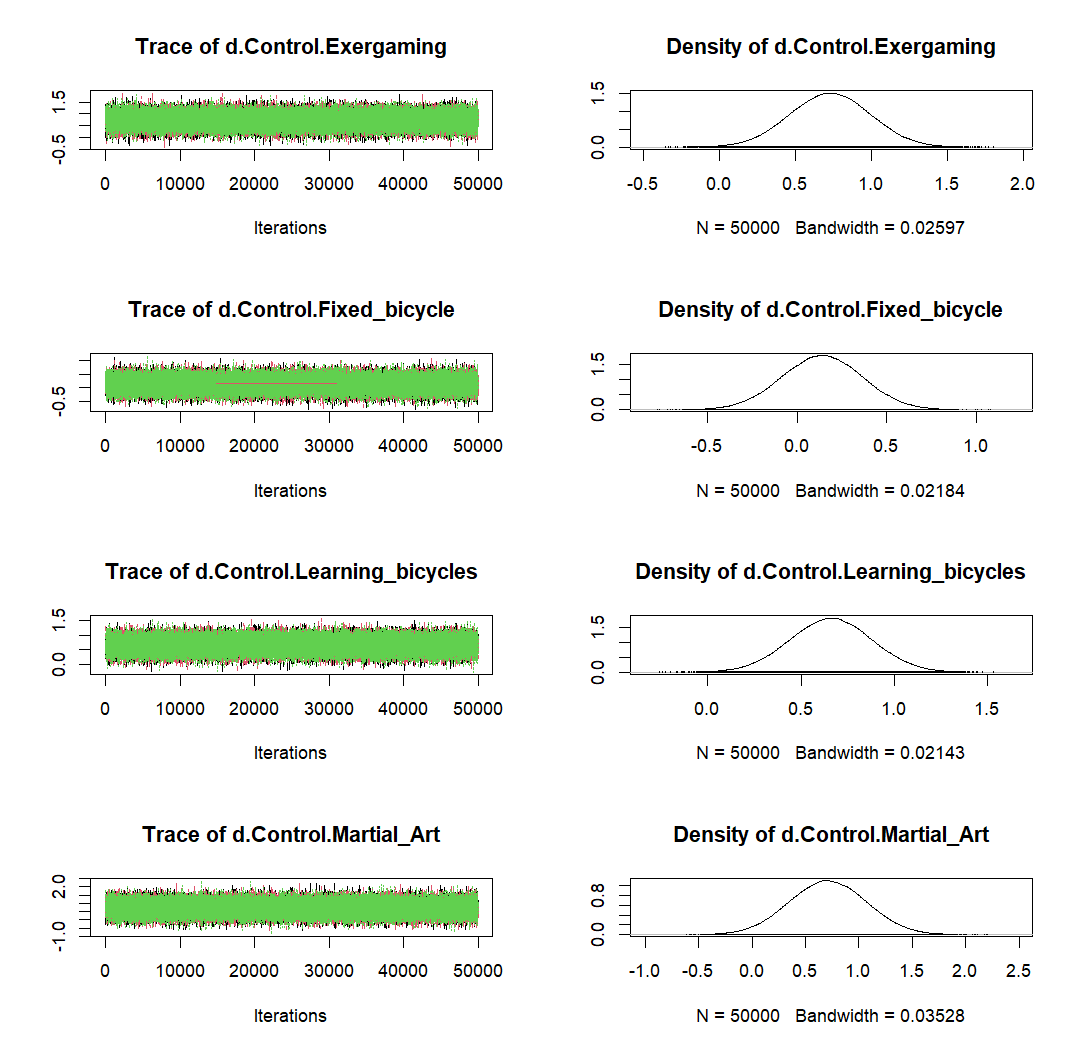

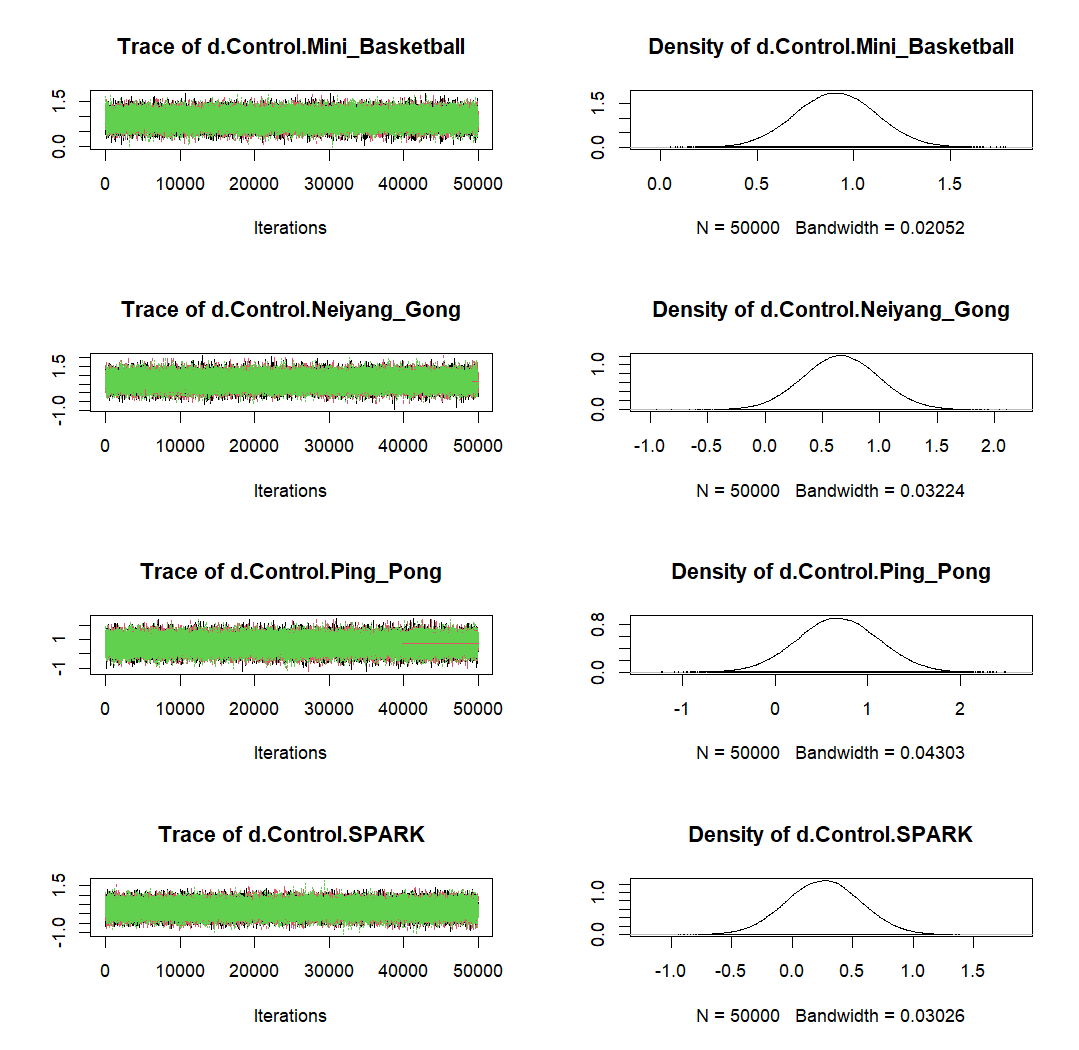


**Figure 9** Consistency Model and Inconsistency Model of Inhibition Control


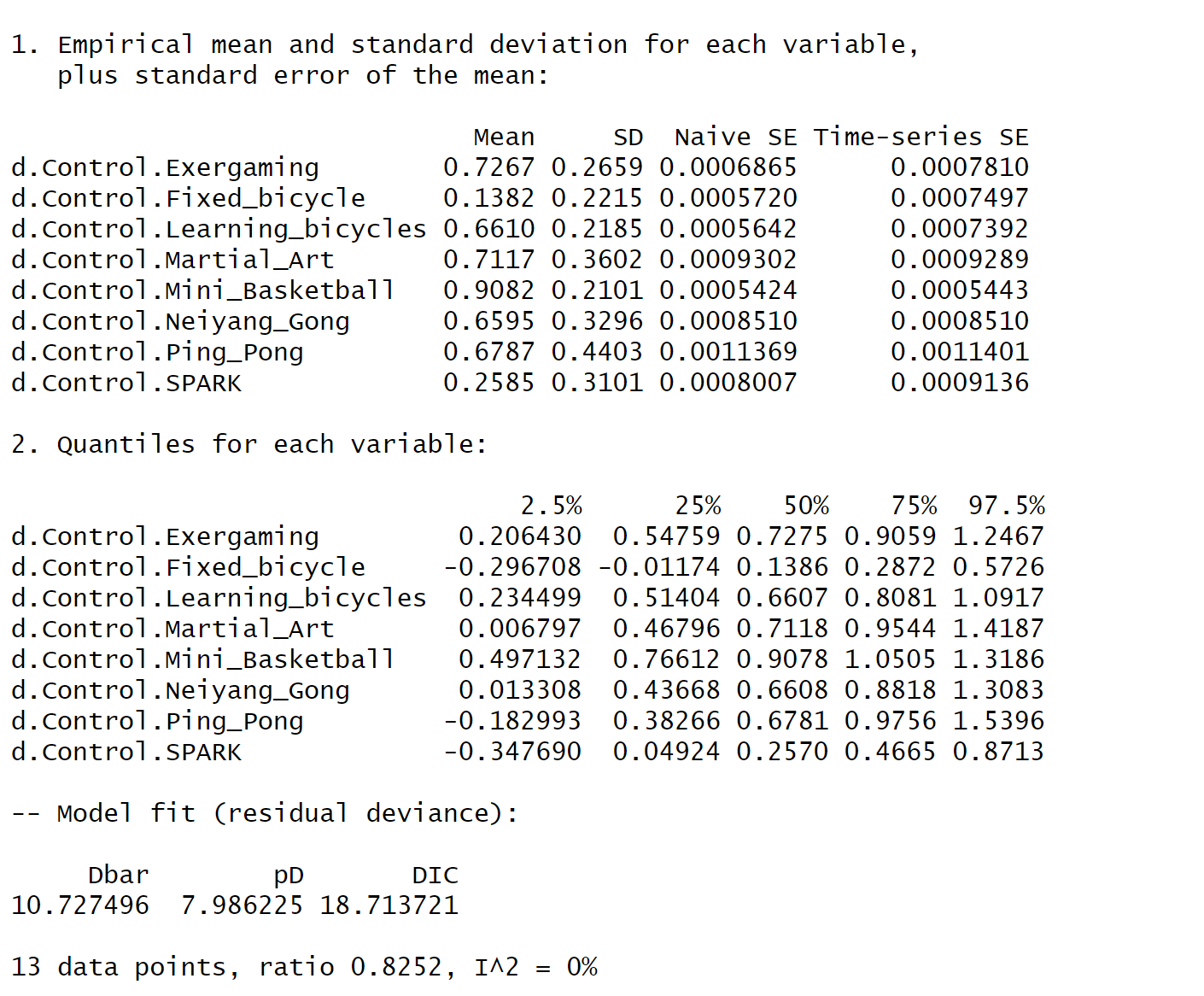


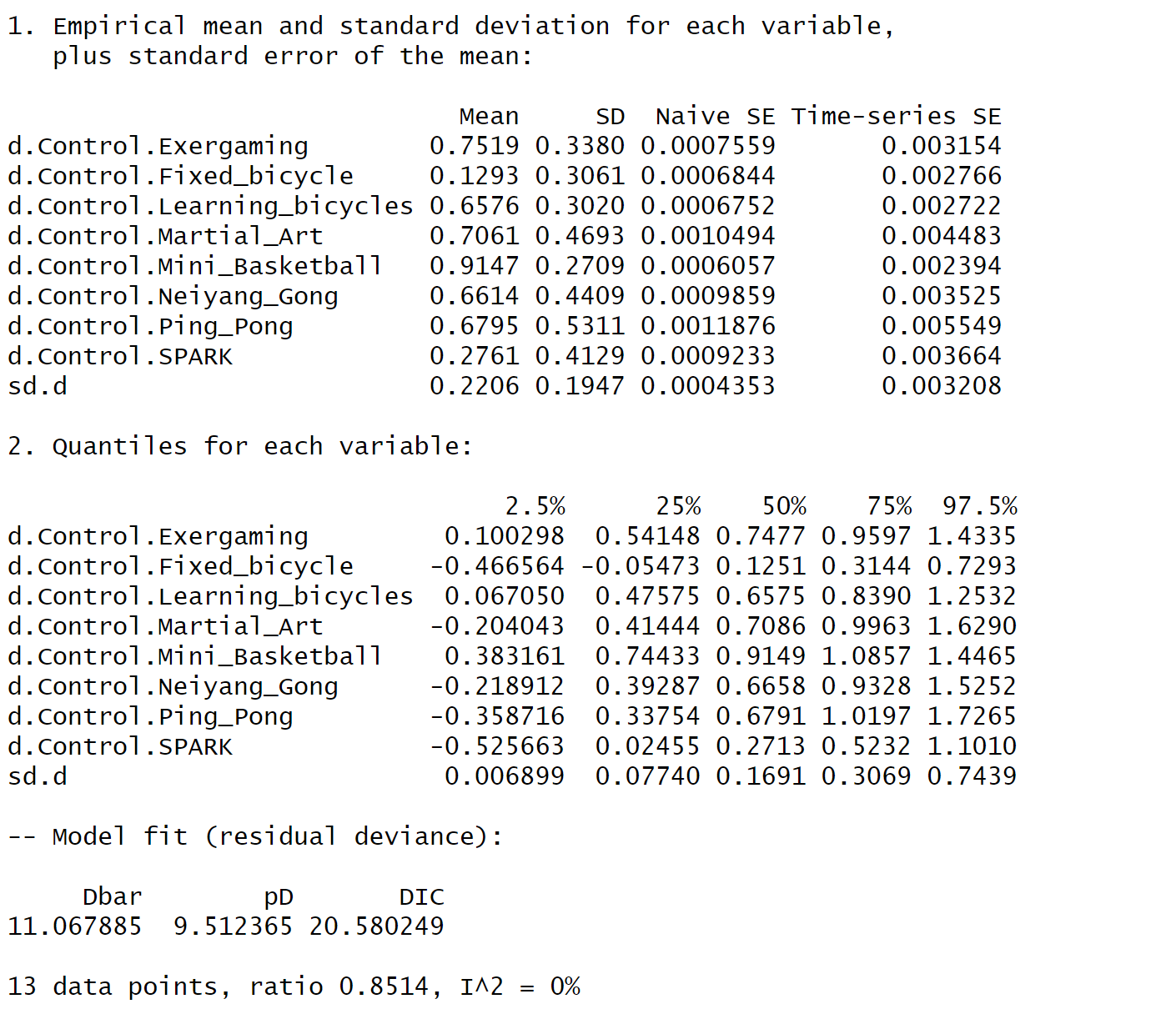


**Figure 10** Cumulative Ranking Plot of Intervention Measures on Dimensions of Inhibition Control


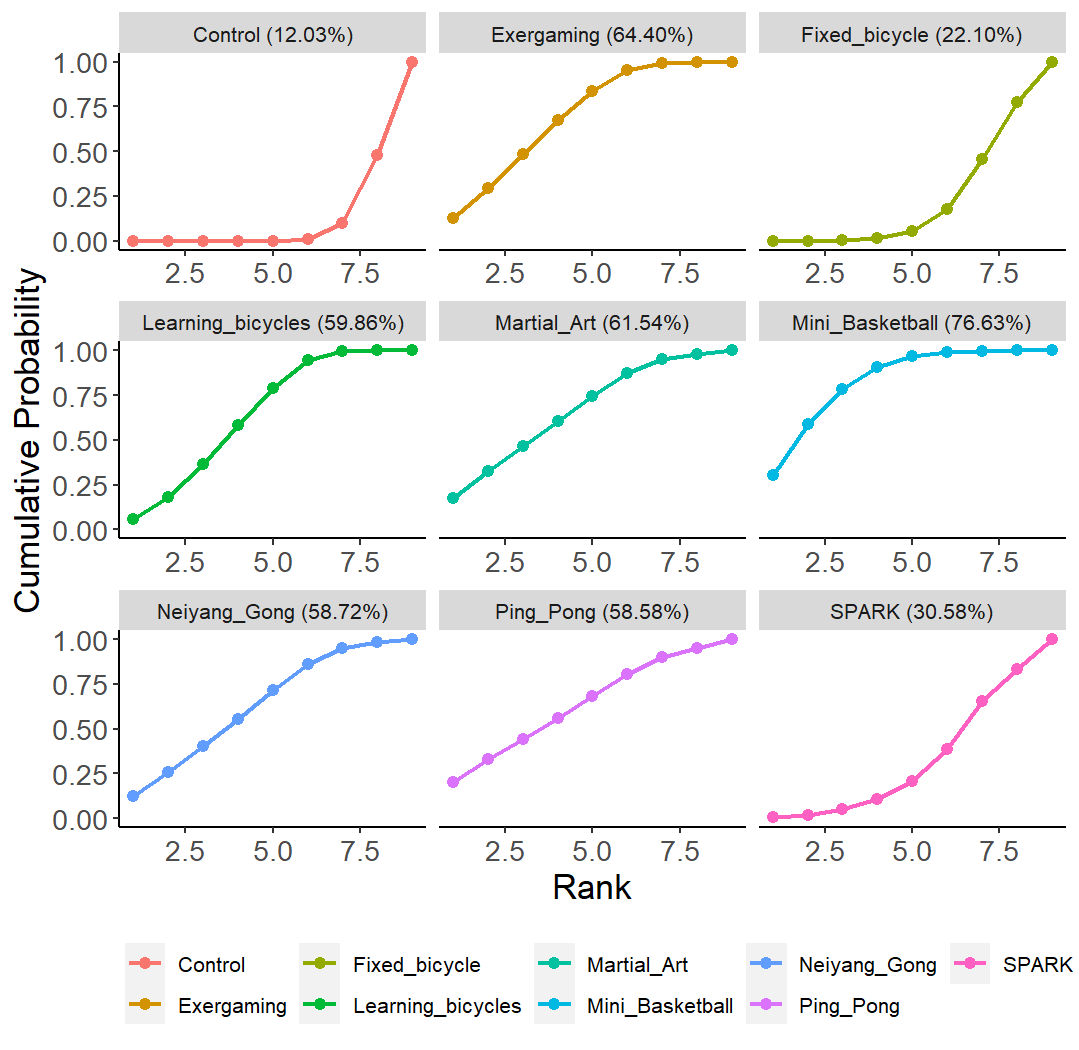


**Figure 11** Funnel Plot of Inhibition Control Dimensions


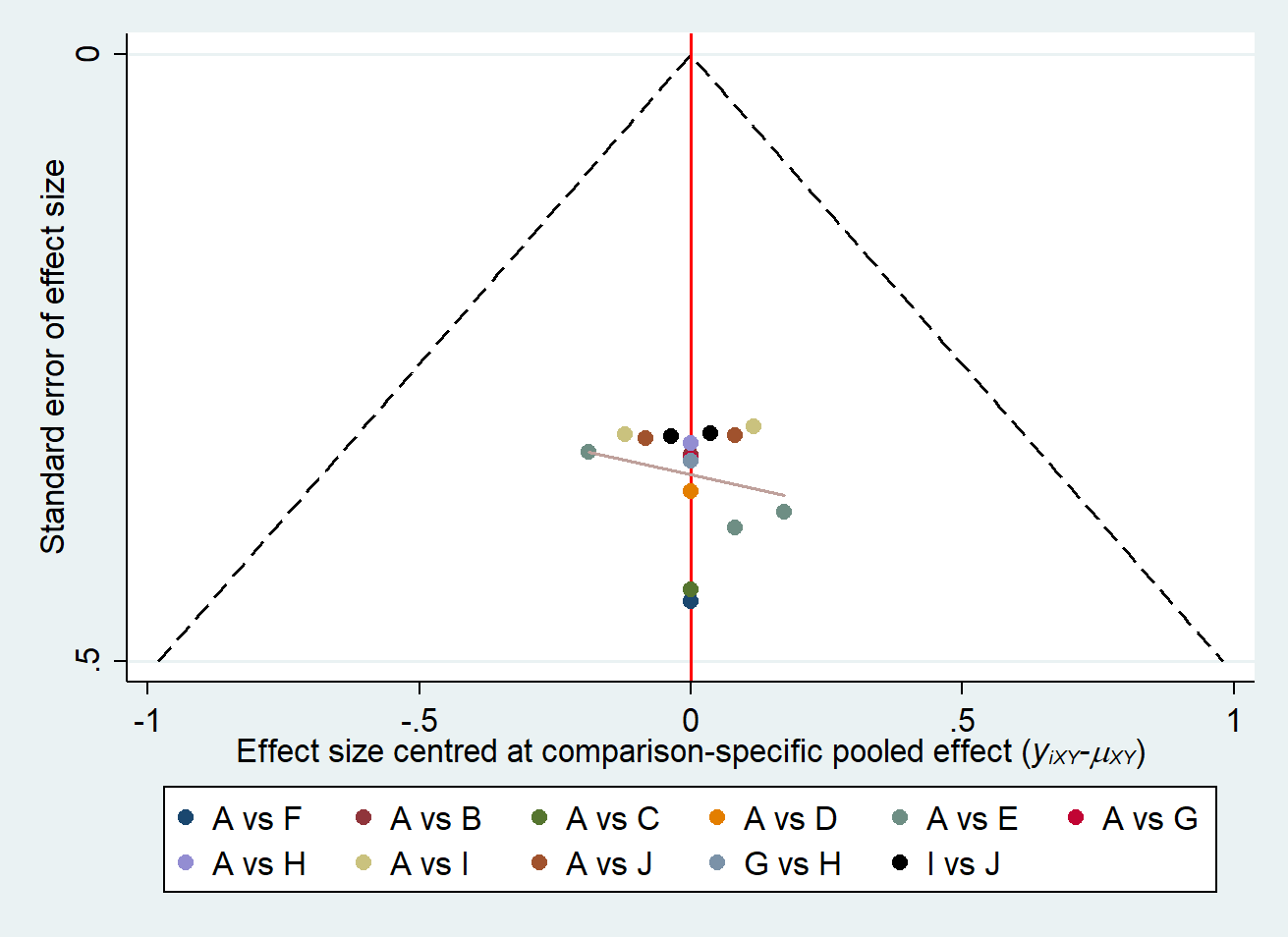


**Figure 12** Meta-Regression Results of Inhibition Control


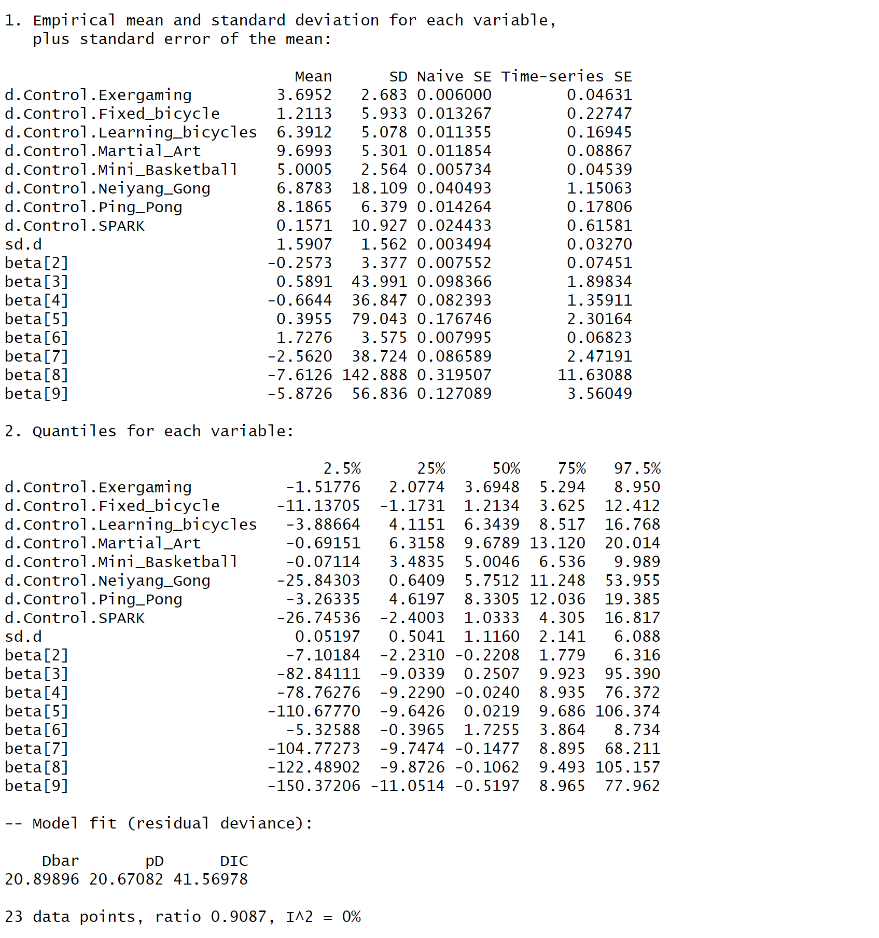

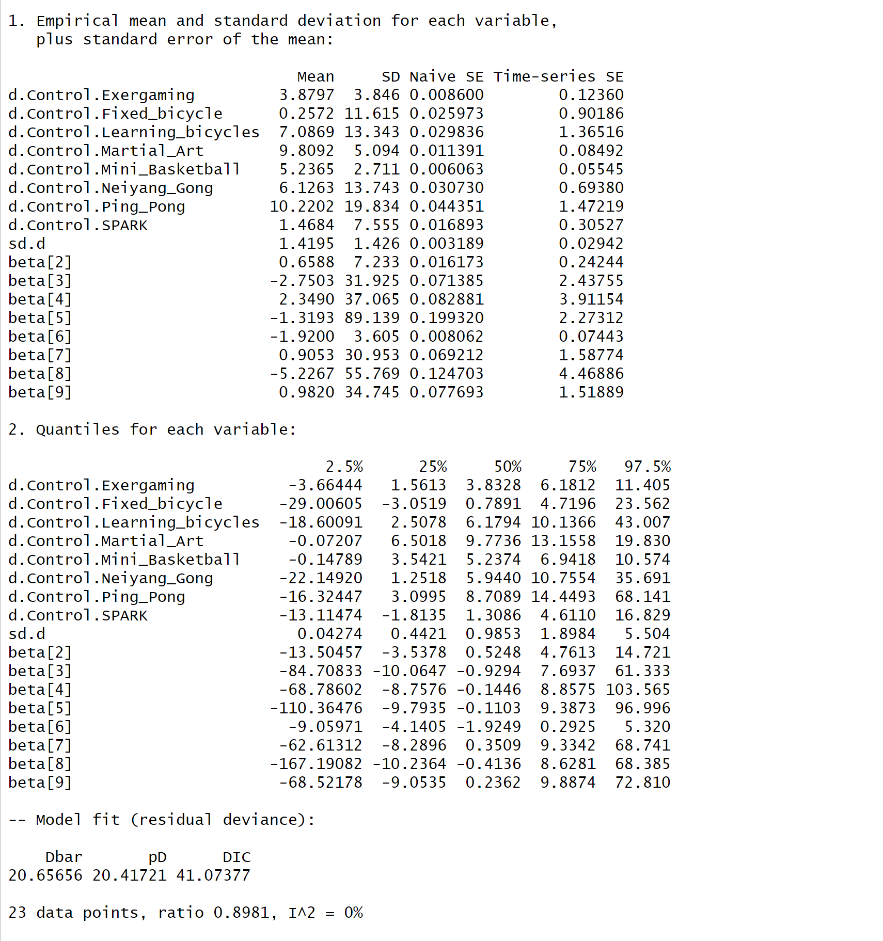


Average Age Total duration/hour


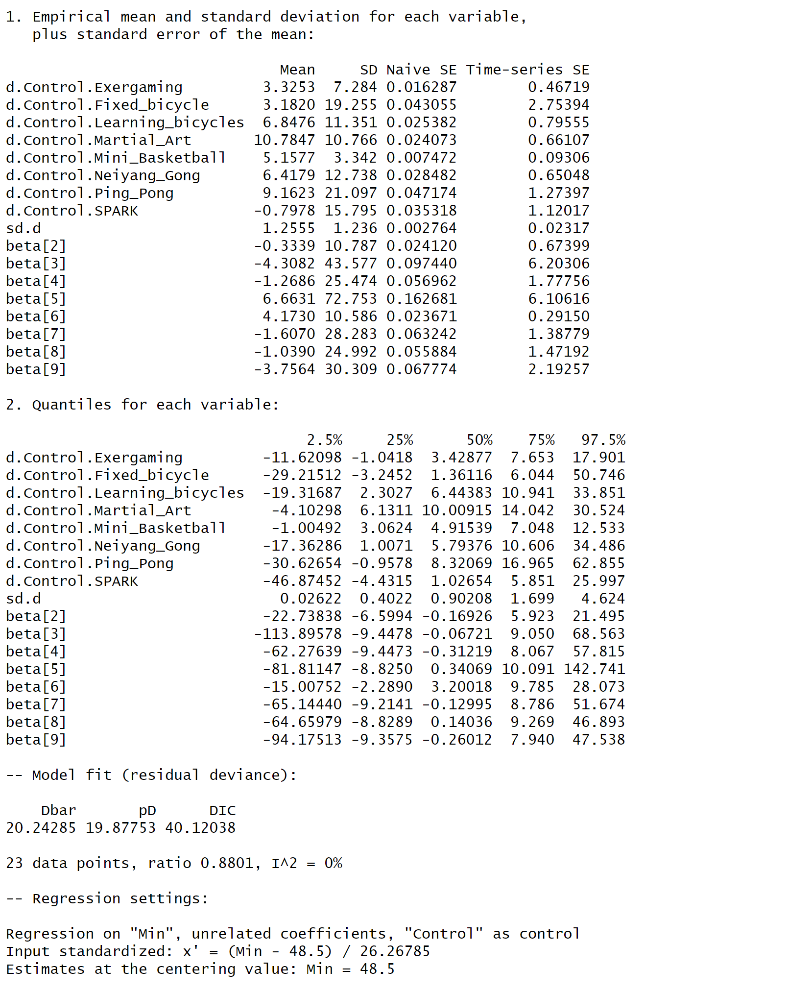

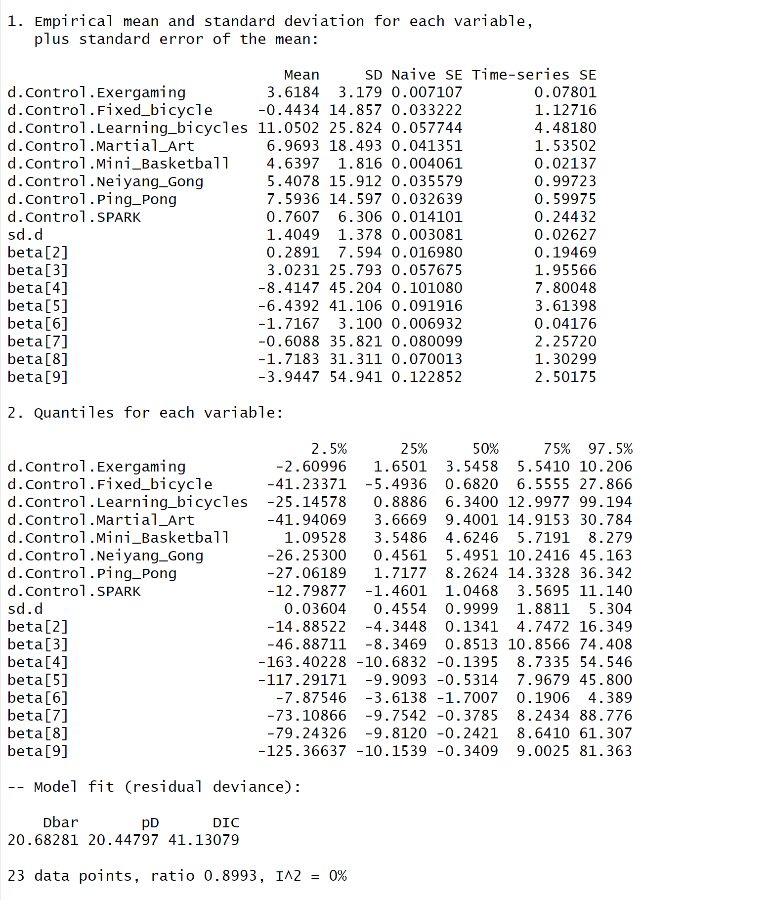


Duration per minute Weekly intervention frequency


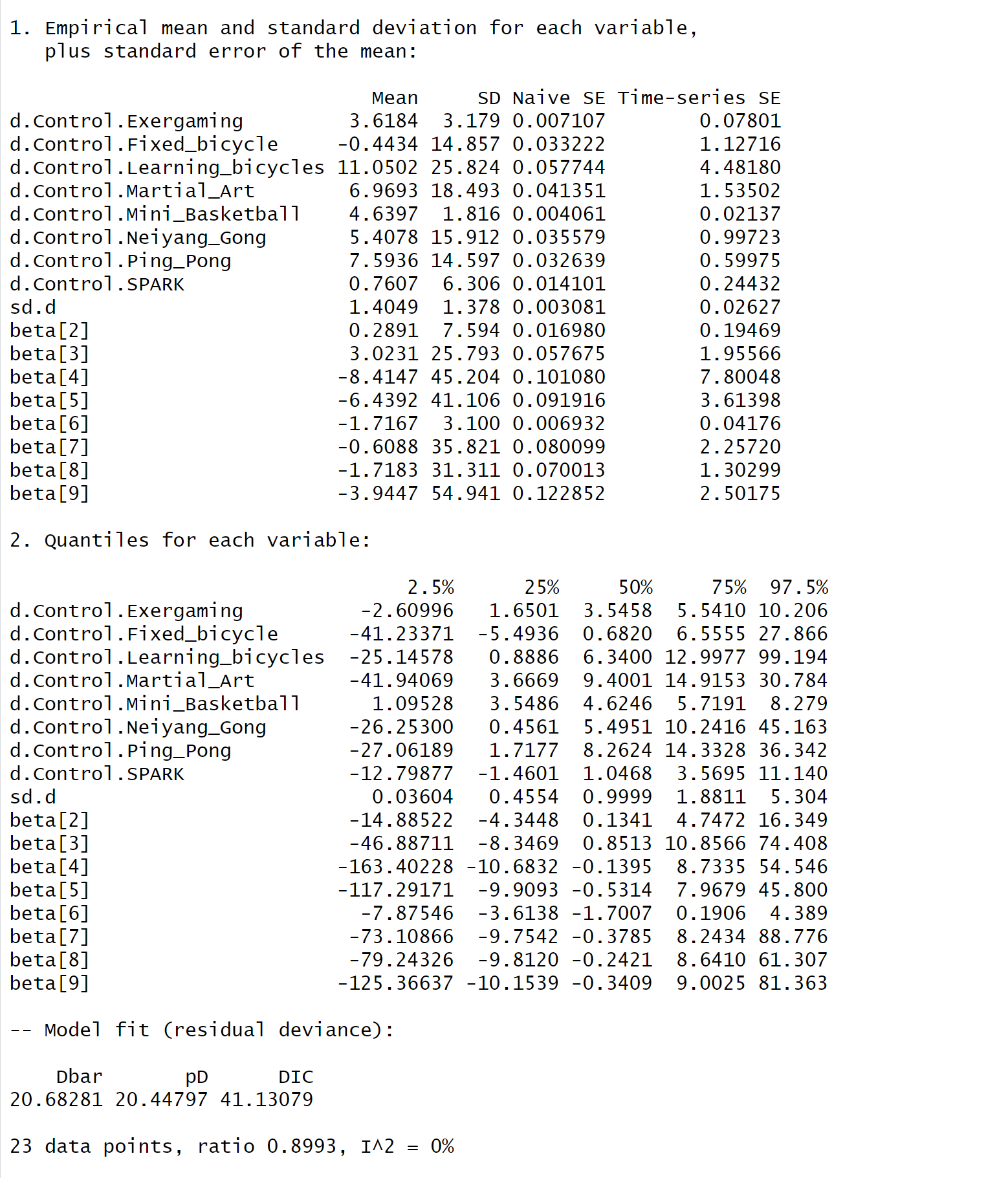

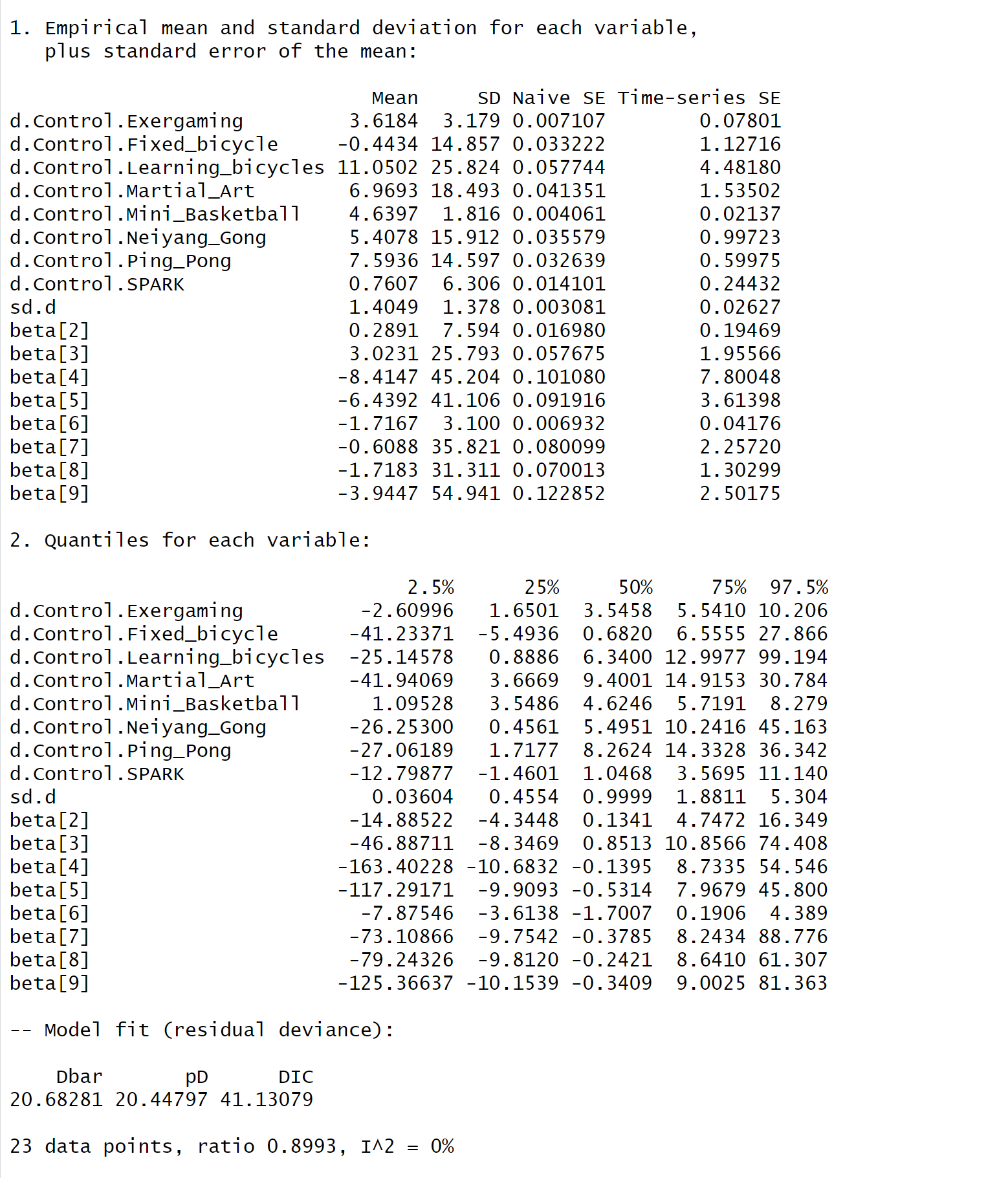


Duration Week Year of publication

## Working Memory

**Figure 13** Convergent Diagnostics of Working Memory


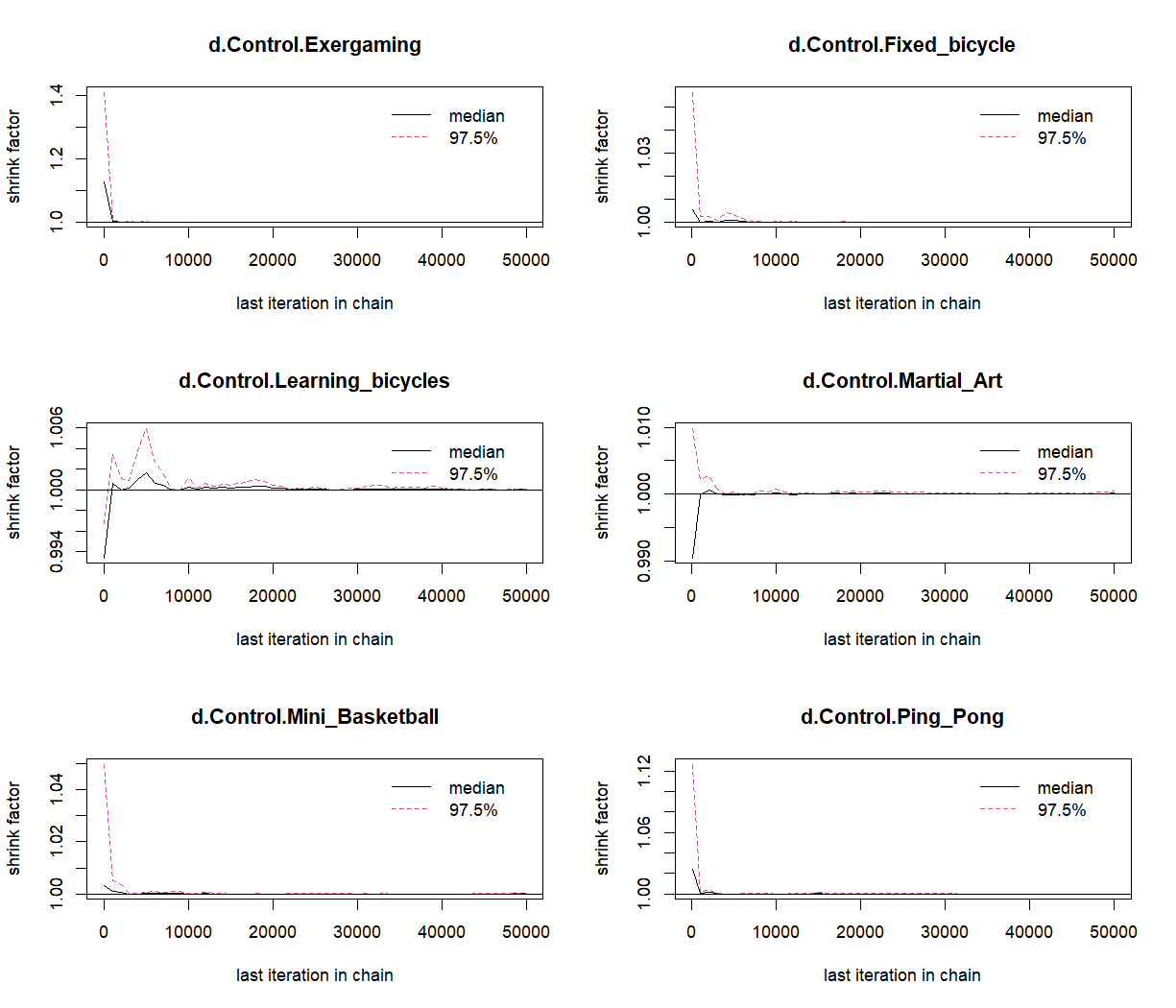


**Figure 14** Trajectory and Density Plots of Various Interventions on Dimensions of Working Memory
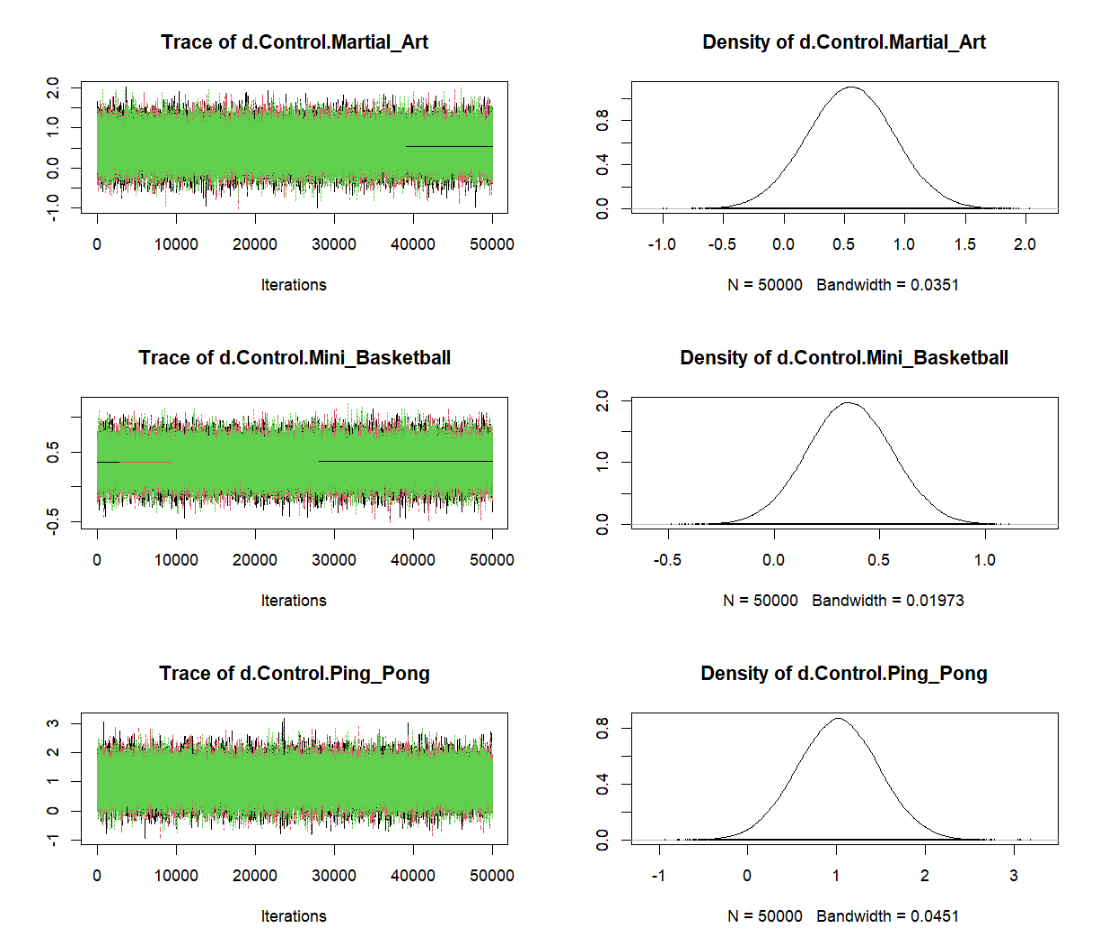

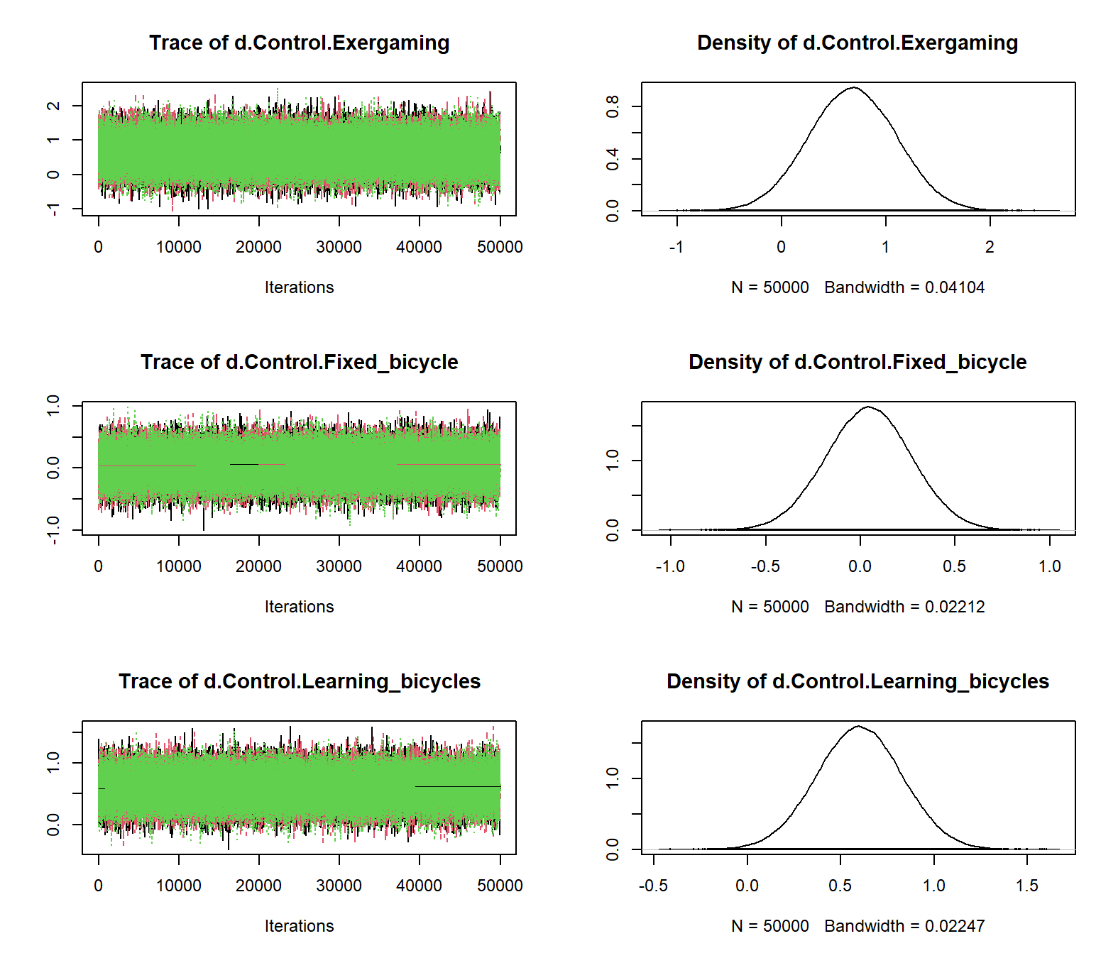


**Figure 15** Consistency Model and Inconsistency Model of Working Memory


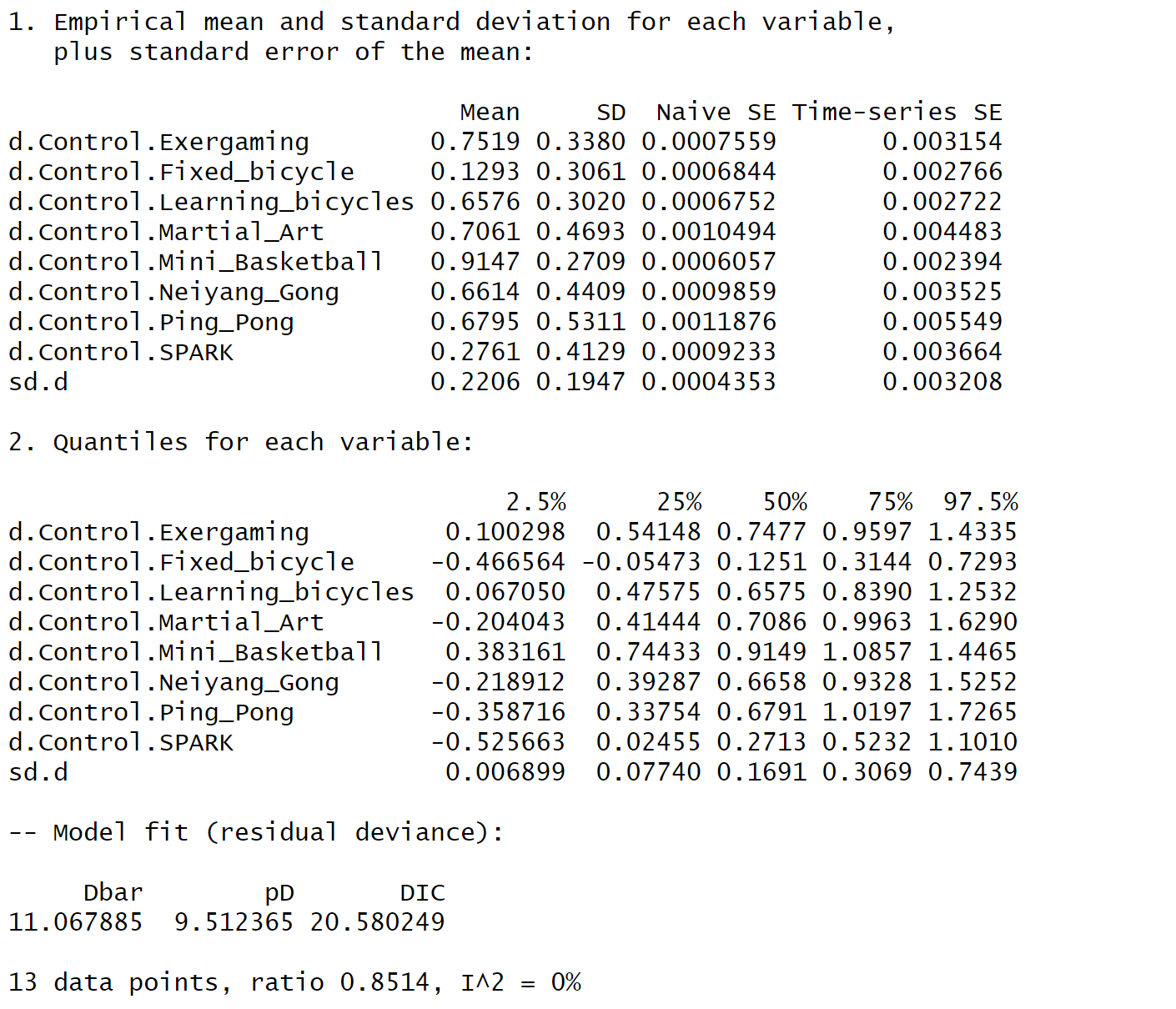


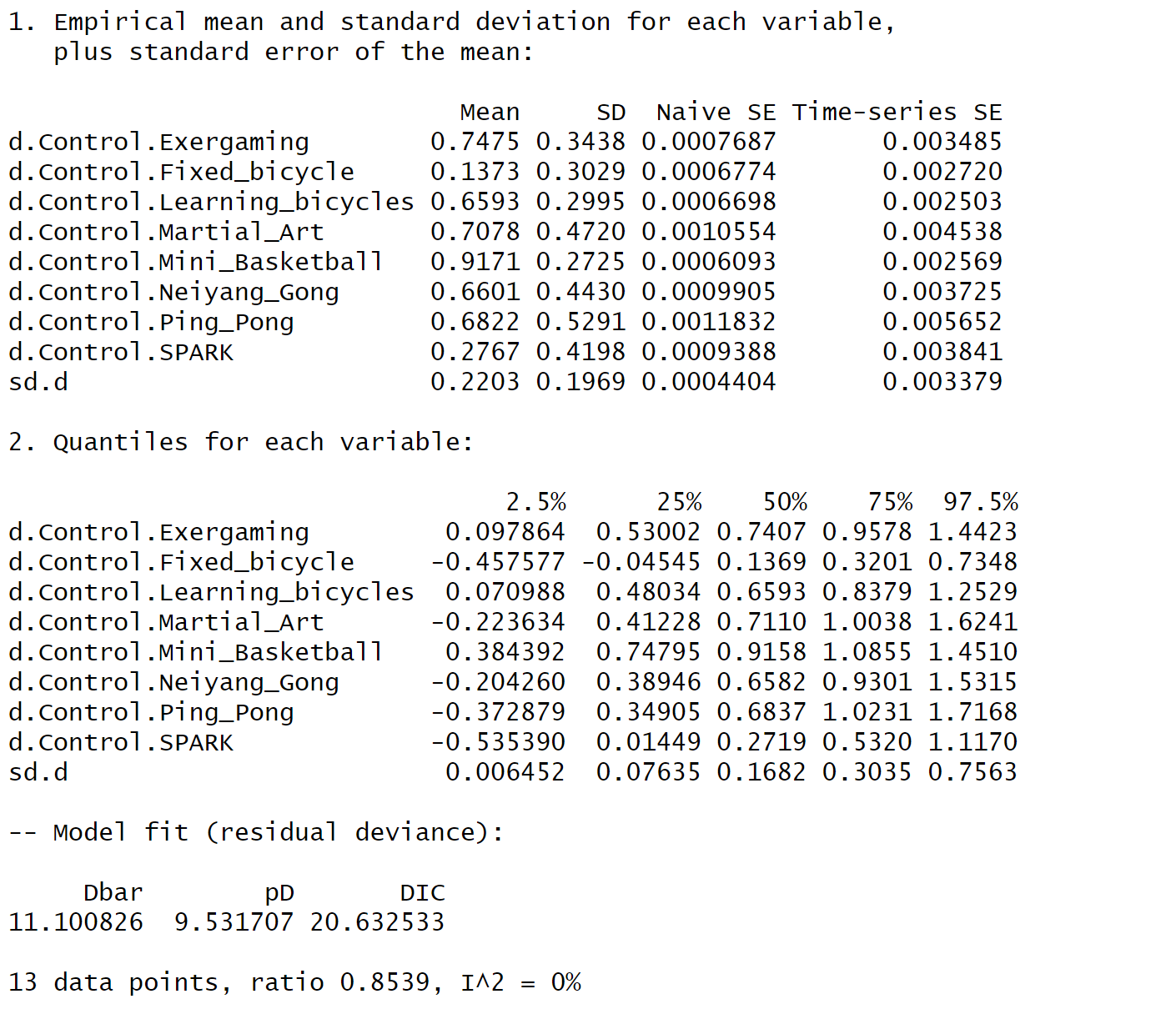


**Figure 16** Cumulative Ranking Plot of Intervention Measures on Dimensions of Working Memory


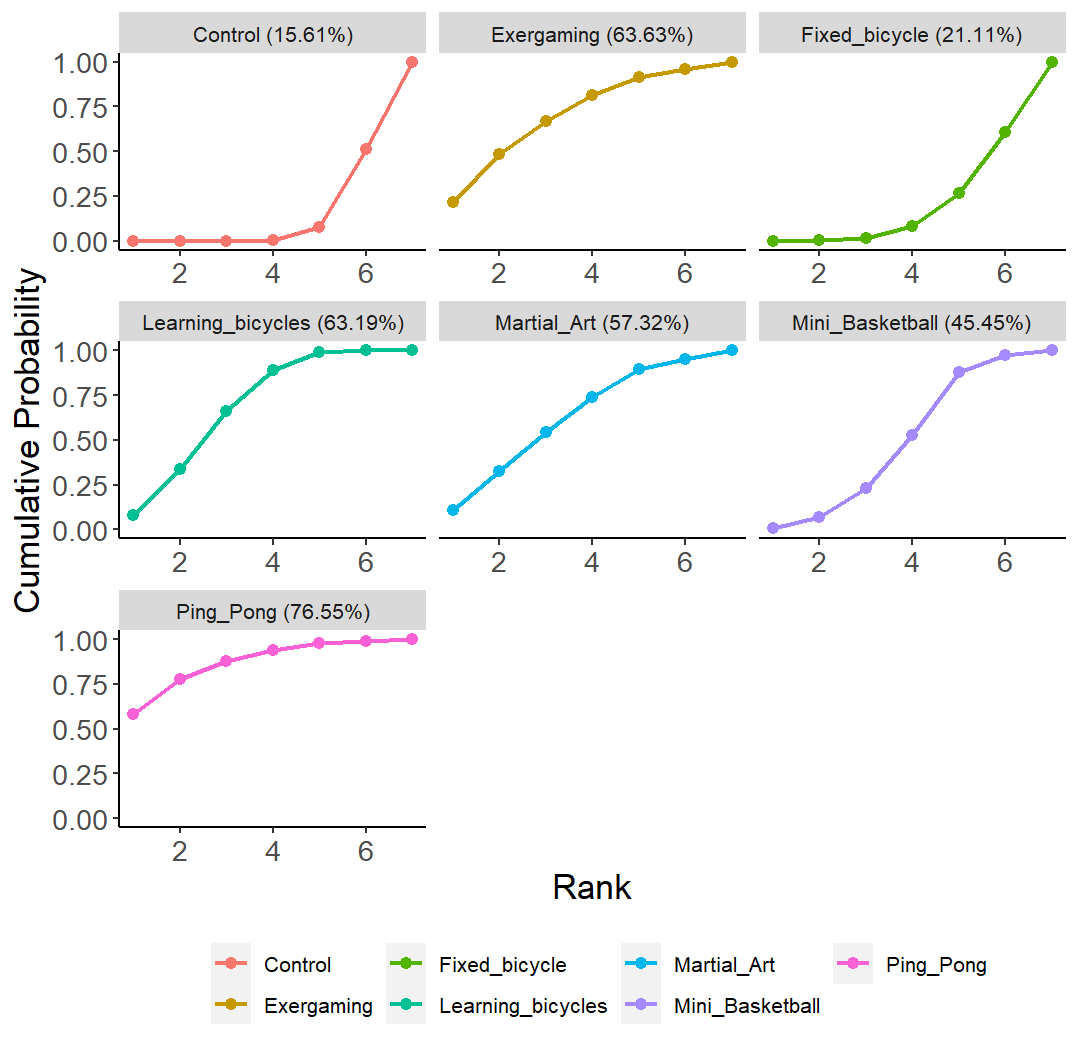


**Figure 17** Funnel Plot of Working Memory Dimensions


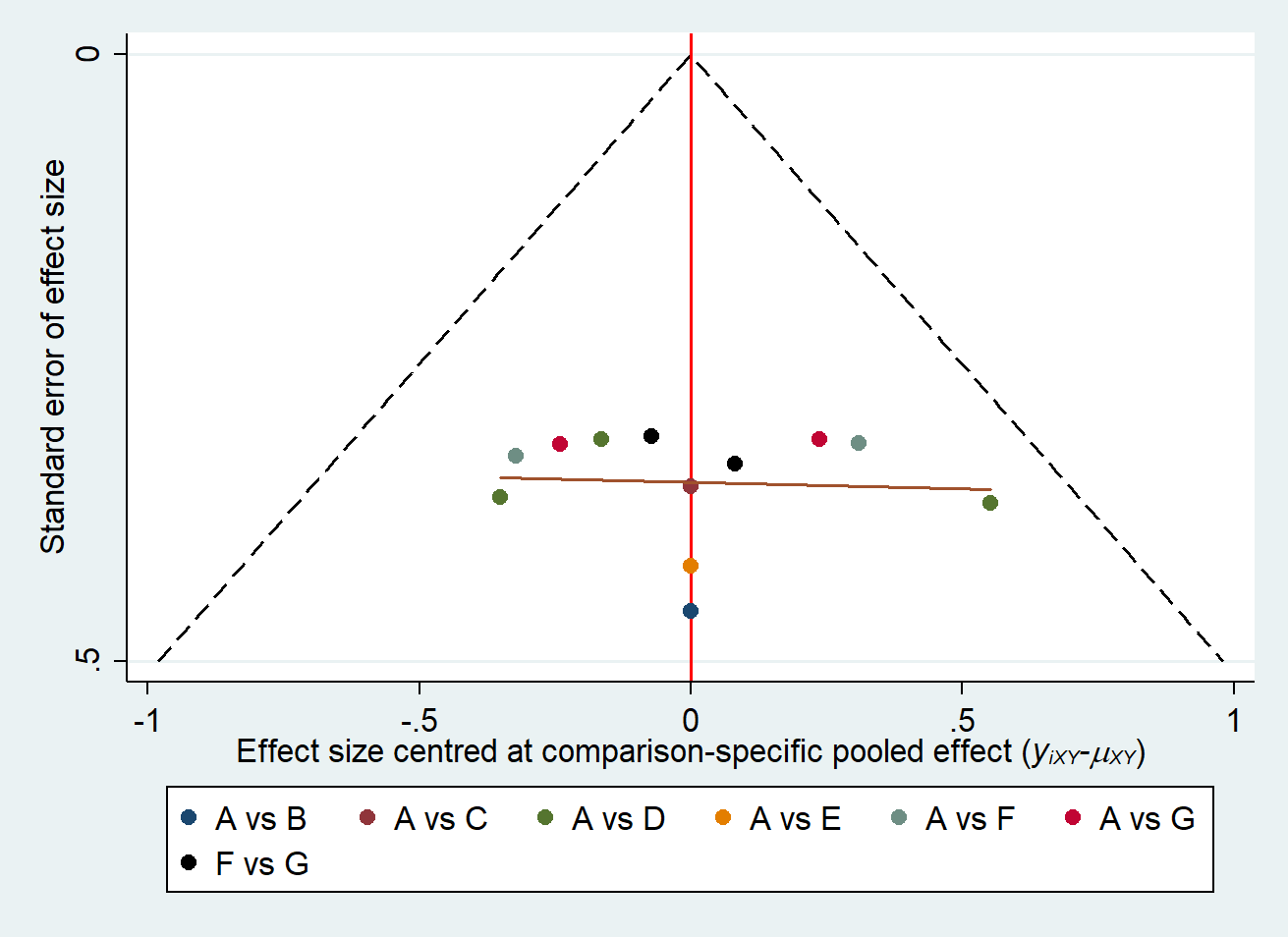


**Figure 18** Meta-Regression Results of Working Memory


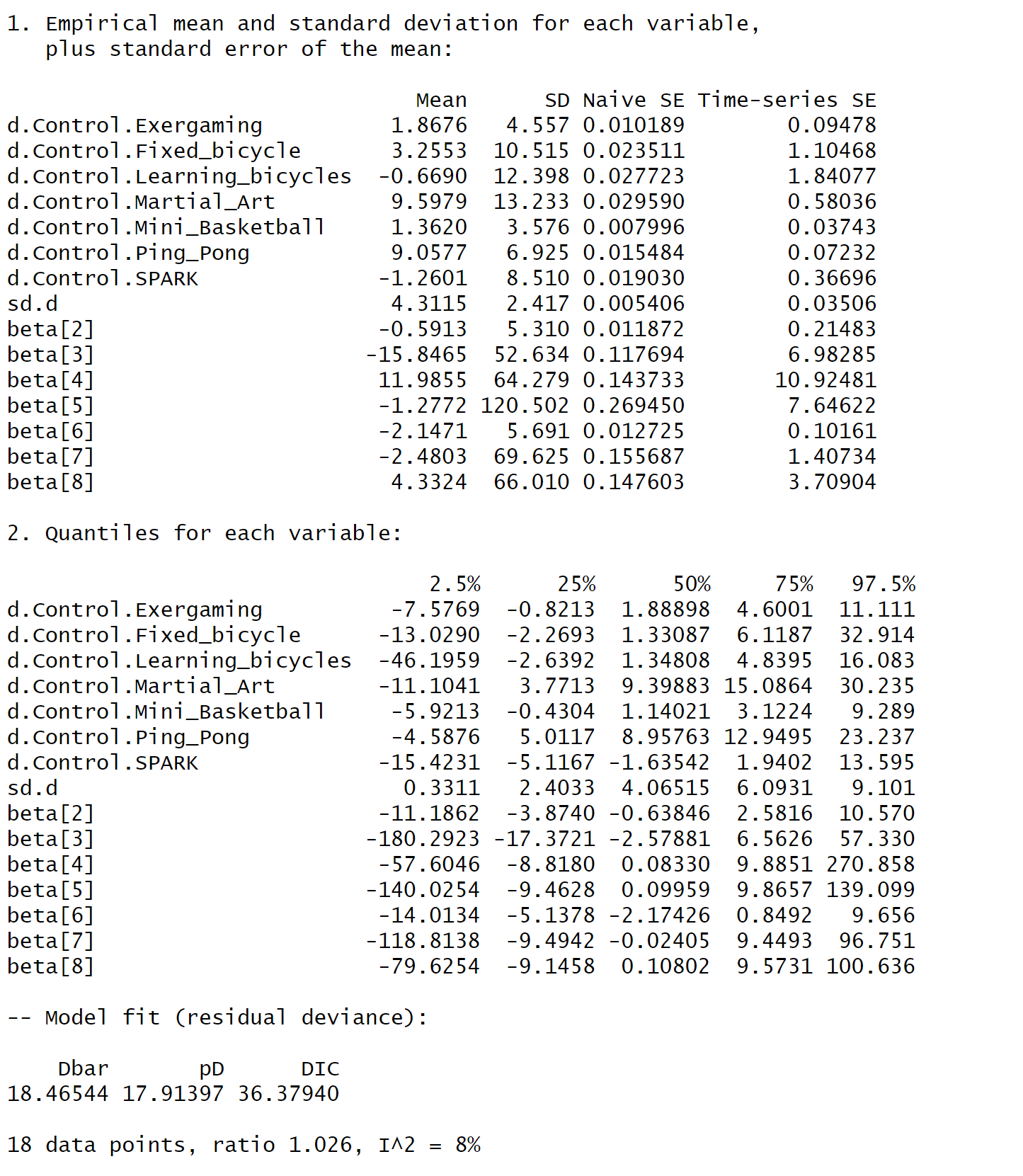

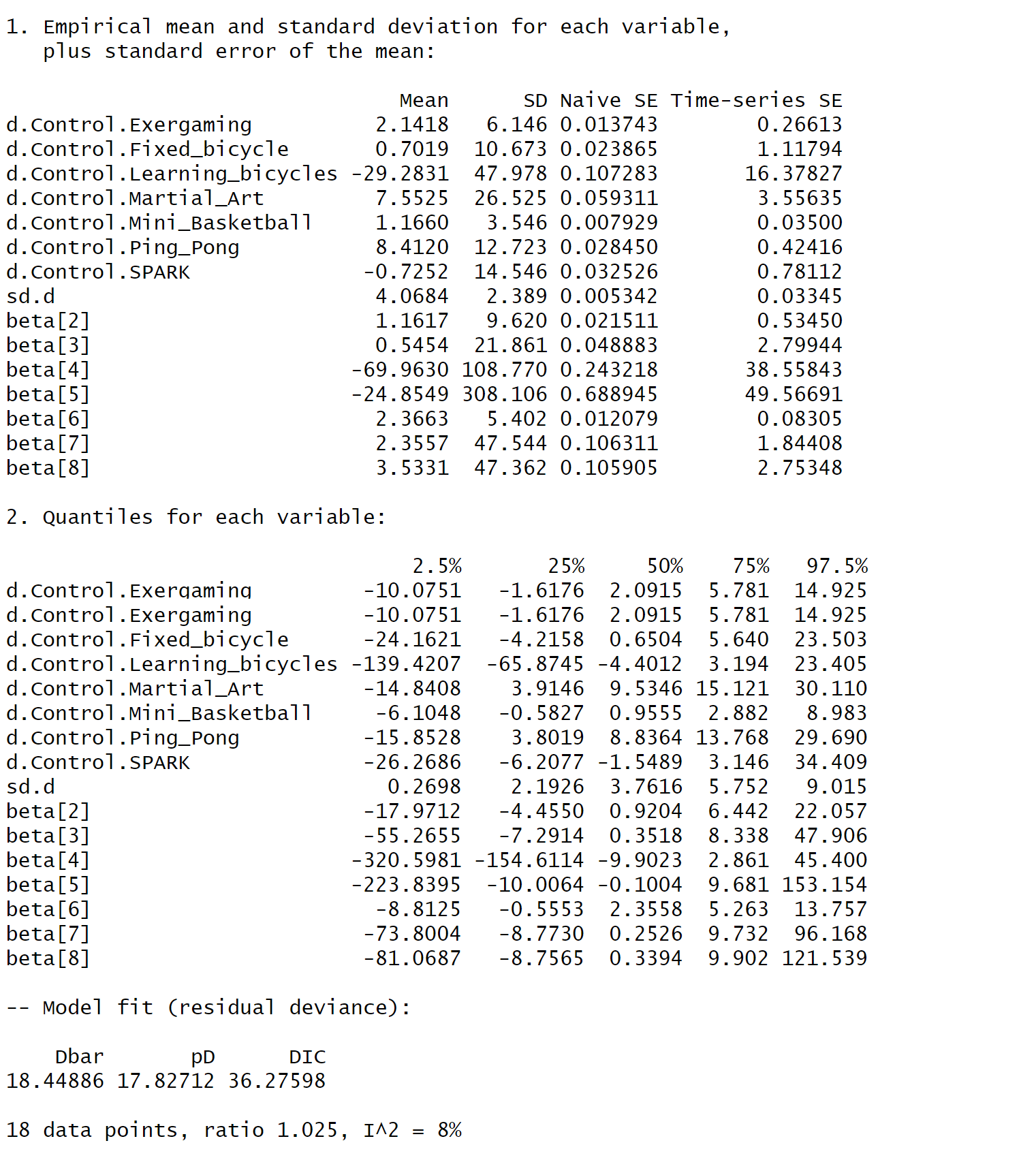


Average Age Total duration/hour


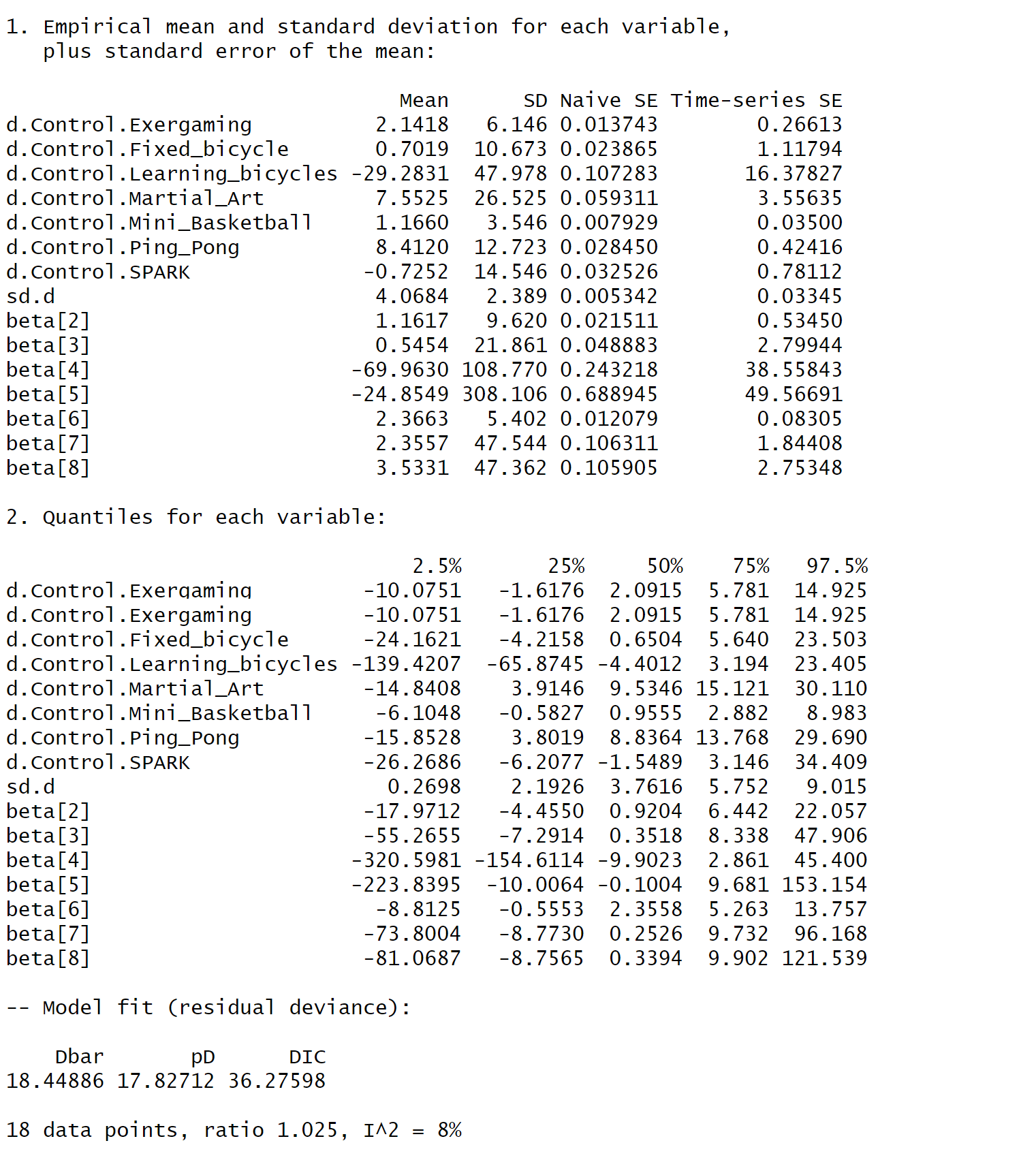

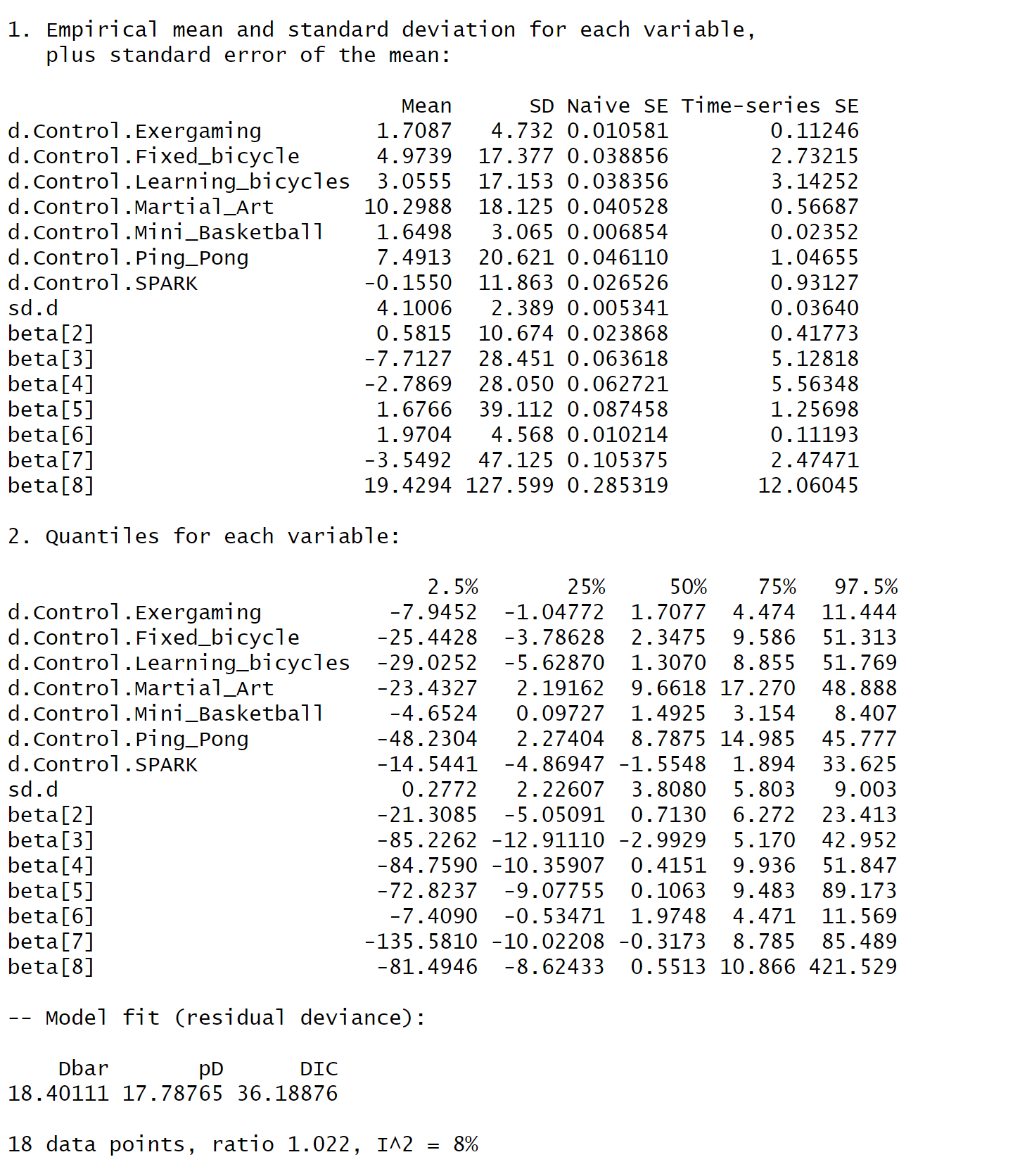


Duration Per Minute Weekly intervention frequency


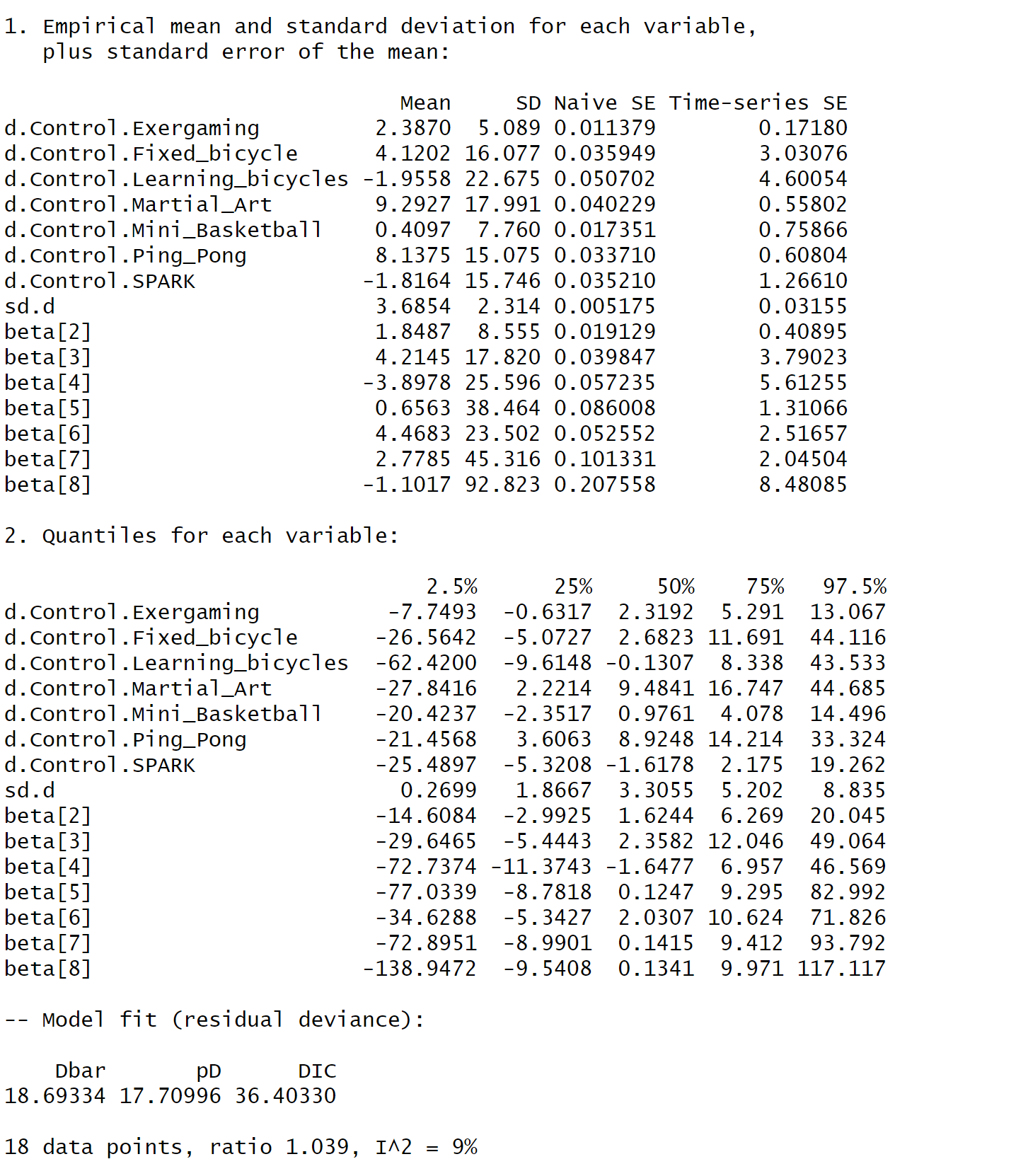

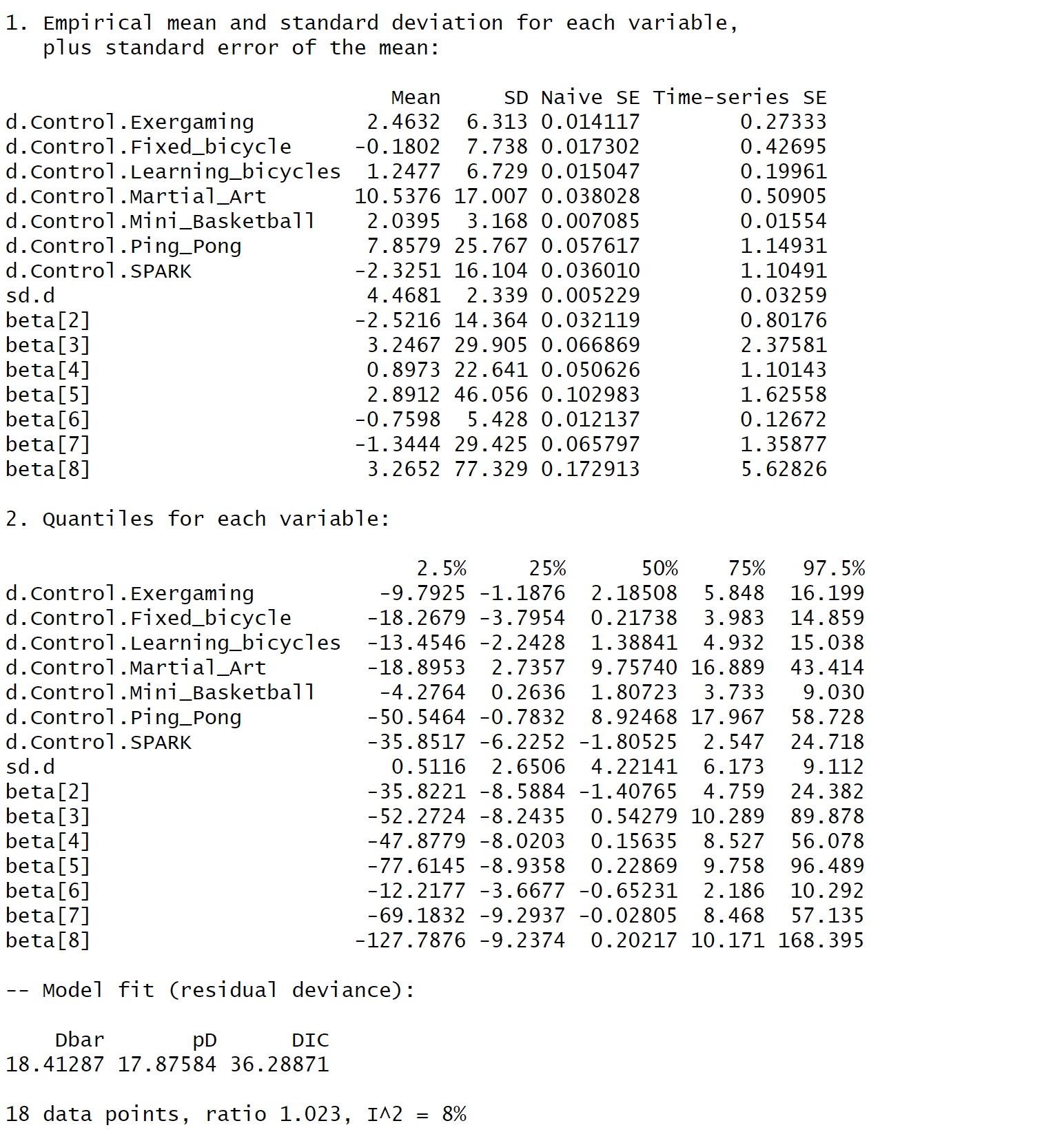


Duration Week Year of publication
